# Application of Infrared and Near-Infrared Microspectroscopy to Microplastic Human Exposure Measurements

**DOI:** 10.1177/00037028231199772

**Published:** 2023-10-04

**Authors:** Stephanie Wright, Joseph Levermore, Yukari Ishikawa

**Affiliations:** 1 Environmental Research Group, School of Public Health, Imperial College London, London UK; 2 MRC Centre for Environment and Health, School of Public Health, Imperial College London, London UK; 3 NIHR Health Protection Research Unit in Environmental Exposures and Health, School of Public Health, Imperial College London, London UK

**Keywords:** Microplastic, Raman spectroscopy, Fourier transform infrared spectroscopy, FT-IR, IR spectroscopy, human exposure

## Abstract

Microplastic pollution is a global issue for the environment and human health. The potential for human exposure to microplastic through drinking water, dust, food, and air raises concern, since experimental in vitro and in vivo toxicology studies suggest there is a level of hazard associated with high microplastic concentrations. However, to infer the likelihood of hazards manifesting in the human population, a robust understanding of exposure concentrations is needed. Infrared and near-infrared microspectroscopies have routinely been used to analyze microplastic in different exposure matrices (air, dust, food, and water), with technological advances coupling multivariate and machine learning algorithms to spectral data. This focal point article will highlight the application of infrared and Raman modes of spectroscopy to detect, characterize, and quantify microplastic particles, with a focus on human exposure to microplastic. Methodologies and state-of-the-art approaches will be reported and potential confounding variables and challenges in microplastic analysis discussed. The article provides an up-to-date review of the literature on microplastic exposure measurement using (near) infrared spectroscopies as an analytical tool, highlighting the recent advances in this rapidly advancing field.

## Graphical abstract

**Graphical Abstract:**
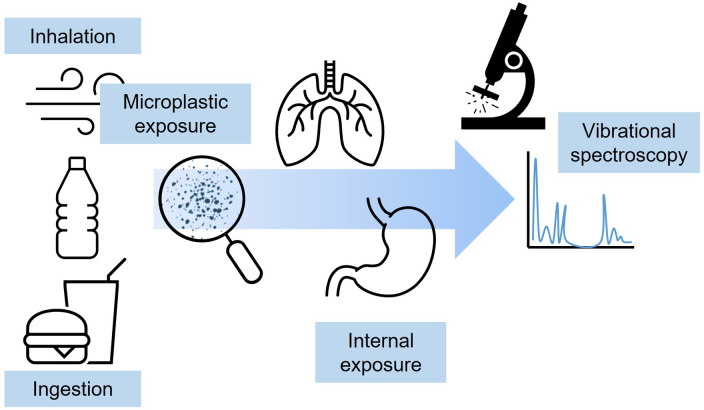
This is a visual representation of the abstract.

## Introduction

### Microplastic Sources, Transport, Pathways, and Fate

The pollutant classes “microplastic” and “nanoplastic” define a heterogeneous group of particles that are comprised of heavily modified synthetic organic polymers. These particles span a vast size range, measuring 1 µm to 5 mm, and 100 nm to 1 µm for micro- and nanoplastics, respectively, and are emitted from semisynthetic plastic materials following degradation via several pathways, predominantly exposure to ultraviolet and/or mechanical energy. The variety of shapes in which they occur, although mainly as fragments or fibers, reflects the diverse range of microplastic sources, owed to the versatility and widespread industrial applications of plastic.^1^ It is unknown whether micro and nanoplastic and plastic nanoparticles (<100 nm) ever fully remineralize in the environment and thus are considered persistent, with the potential to accumulate. Over the last two decades, experimental laboratory-based studies have highlighted the potential for certain microplastic particles to adversely impact metrics of growth,^2^ survival,^3^ and reproduction^4^ in biota. As the production volumes of plastic, the source of microplastic, continue to grow,^5^ there are concerns that environmental concentrations capable of negatively impacting biota and ecosystems will be reached.^6^

Hypothesized predominant sources of microplastic release to the environment include synthetic textiles (clothing, carpets, and upholstery), tires, fishing gear, and single-use plastic packaging.^1^ In addition to these point sources, there are anthropogenic activities that lead to their release, such as laundering clothes or driving cars, and anthropogenic and environmental processes, which mobilize microplastic pollution to other environmental compartments. An anthropogenic example is the application of contaminated sludge from wastewater treatment facilities, in which over 90% of the microplastic in influent is retained, to agricultural land as a soil conditioner.^7^

Microplastic pollution has been observed in all environmental compartments, from deep ocean sediments^8^ to freshwater systems,^9^ to the atmosphere,^10^ and Arctic Sea ice.^11^ There are many pathways and processes by which microplastic particles may reach and transgress these environments, such as via aerosolization at the sea–air interface (ocean > atmosphere),^12^ deposition (atmosphere > land surface),^13^ runoff (land surface > aquatic environment),^14^ and sedimentation (water column > sediment).^8^ While individual particles may undergo different trajectories once in the environment, it is hypothesized that burial in sediments and soils forms the end of the “microplastic cycle”. Concentrations can be as high as 1.3 × 10^6^ microplastic particles/m^3^ for the marine water column^15^ and 4.1 × 10^5^ microplastic particles/kg for terrestrial soils.^6^

### Human Exposure to Microplastic

As microplastic particles are generated, mobilized, or cycle through the environment, there is potential for human exposure. Following initial early observations of microplastic in the marine environment, studies on the contamination of seafood followed. First focusing on shellfish (bivalve mollusks), then fish, evidence suggests that lower trophic species in contaminated areas ingest microplastic, which thus enters the food chain from an environmental source. Observations of commercial species or specimens purchased from a shop or market highlight the potential for human consumption.^16^ Additionally, other sources of contamination in food systems include packaging, and the factory environment (workers, air, and machinery components). For example, packaged chicken was found to be contaminated with polystyrene (PS) microparticles (4.0–18.7 kg^−1^ meat), hypothesized to originate from the PS tray it was packed in.^17^ Bottled water was contaminated with microplastic particles in addition to the materials comprising the bottle, i.e., polyethylene terephthalate (PET) and polypropylene (PP), possibly from the factory atmosphere, workers’ clothes, or machine parts.^18^ Microplastic has been observed in other beverages, including tap water,^19^ beer,^20^ energy drinks,^21^ milk,^22^ and soft drinks.^21,23^ The water and ingredients could also be sources of microplastic. The highest exposures seem to come from packaging sources when high temperatures are involved, i.e., rinsing with hot water.^24^

A variety of food groups have been investigated for the contamination of microplastic. While the number of studies on shellfish and fish predominate, salt, honey, milk, and rice have also been studied.^1^ Key food groups, such as cereals and grains, roots, fruits, and vegetables remain understudied yet comprise a large proportion of the global population's diet. Probabilistic modeling of the published data results in a conservative estimated total daily median microplastic mass intake of 0.2 (0.0001–7500) μg child^−1^ day^−1^ and 0.6 (0.0003–17 000) μg adult^−1^ day^−1^ based on data for fish, mollusks, crustaceans, tap water, bottled water, salt, beer, milk, and air.^25^ The contamination of dust^26–28^ and soils^29^ also highlight the potential for passive exposure, either due to deposited dust on foods and beverages, or passive ingestion from hand contact.

In addition to exposure through ingestion due to contaminated dietary sources, microplastic inhalation due to contaminated air may occur. Microplastic contaminates the indoor and outdoor atmospheric environments, with outdoors ranging from urban to remote pristine to open ocean atmospheres.^10^ Of concern are airborne microplastic particles with an aerodynamic diameter of <10 µm and 2.5 µm, as these may lead to exposure in the central and distal regions of the human airways, respectively. Evidence on this is sparse. Levels may reach ∼2500 microplastic particles/m^3^ in the 10 µm fraction of airborne particulate matter in urban traffic environments,^30^ although this should be interpreted with caution due to the lack of data.

Several studies have recently been published on microplastic in human tissues, as further confirmation of exposure, and potentially indicating biodistribution in the body following exposure as well as points of accumulation. Tissues studied include breast milk,^31,32^ placenta,^32–34^ meconium,^32,34^ stool,^35,36^ colon,^37,38^ blood,^39^ lung,^40,41^ and cerebrospinal fluid.^42^ However, often these studies are on processed (digested) tissue, with an opportunity for laboratory background contamination of samples to occur;^43^ potential for sample contamination from the surgical environment^44^ is often not considered; and the particle sizes observed mostly do not follow biological plausibility based on what is known for particle kinetics and distribution in the body, hence more research is needed.

### Potential Health Effects Due to Microplastic Exposure

With exposure through ingestion and inhalation likely, the impact that microplastic may have on population health is a concern. Epidemiological studies are currently lacking, however, past occupational epidemiology studies indicate that inhalation of high levels of respirable plastic dust can lead to chronic inflammation and interstitial lung disease^45–49^ (reviewed by World Health Organization [WHO]).^1^ How this high exposure to a somewhat pure source of microplastic translates to the lower everyday exposures that populations experience, i.e., a mixture of microplastic within a mixture of other particles and chemicals, is unknown. Laboratory-based hazard characterization studies in in vitro models of the gut and lung most commonly observe inflammation and oxidative stress in response to high concentrations of microplastic particles. However, these studies tend to use test particles that are a single type of plastic, i.e., mostly PS, and a single shape, i.e., mostly spherical, in a pristine state (reviewed in WHO^1^). Again, extrapolation to human exposure and the resulting level of hazard is challenging. Finally, there is a growing body of evidence to suggest microplastic ingestion may lead to adverse reproductive and multigenerational effects, although test materials are often under-characterized from the perspective of organic impurities, and thus whether these outcomes are driven by the microplastic particles or chemical additives or contaminants they are carrying is unknown (reviewed in Coffin et al.^50^). As above, these studies focus on a narrow set of microplastic, with little resemblance to the range of microplastic which humans are exposed to.

Thus, data on hazards for microplastic particles that reflect human exposure is needed. In conjunction, robust exposure measurement data, including particle sizes relevant to human physiology, are also needed to help inform the level of risk that these materials present as an environmental pollutant.

### Analytical Methods for Microplastic Classification, Characterization, and Quantification

Plastics are high-molecular-weight substances comprised of synthetic organic polymers, repeating chains of monomeric hydrocarbon units with or without sulfur, nitrogen, or oxygen atoms attached. They are heavily modified through the addition of additives, to give them certain properties, and can be classified as thermoset, e.g., polyurethane (PU), epoxy resin, or thermoplastic, e.g., polyethylene (PE), PP, PS, polyvinylchloride (PVC), PET, and polyamide (PA), depending on how they respond to heat. Plastic can be further classified by type, with the most common plastics produced and encountered in the environment being PE, PP, PET, PVC, and PS. Classifying microplastic particles by type is important, as it indicates potential sources and problematic plastics for targeting policy, regulation, remediation, and engineering. Thus, analytical techniques capable of fingerprinting the composition of micro- and nanoplastic particles are needed.

As early as the term “microplastic” was coined, Fourier transform infrared spectroscopy (FT-IR) was used to classify the spectra of plastic particles.^51^ This IR technique allows for the presence of chemical groups (e.g., C=O, C–H) in a material or substance to be determined, even quantified. This is due to the characteristic frequencies of these groups, at which absorption or transmission of IR (wavelengths between 400 and 4000 cm^−1^) electromagnetic radiation occurs. Through comparison to a library of IR reference spectra, specific absorptions can be assigned to specific groups to confirm the plastic type. There are several variants of FT-IR, from attenuated total reflection (ATR) FT-IR used for larger samples that can be physically handled, to FT-IR coupled with optical microscopy (FT-IR microspectroscopy, or micro-FT-IR). FT-IR has been used to detect and classify microplastic in all matrices indicative of human exposure, from drinking water^52–59^ to the studied food groups,^60–65^ to air,^66–68^ dust,^26,28^ and human tissues and biofluids.^33,34,37,40,44,69–71^

The other vibrational spectroscopy technique that has been applied to microplastic classification, although by less than half as much (based on a PubMed search, “microplastic and FT-IR” resulting in 1102 articles versus “microplastic and Raman” resulting in 494 articles), is Raman spectroscopy. In contrast to the IR spectrum used in FT-IR, Raman spectroscopy irradiates a sample with a monochromatic laser of a defined wavelength, which influences the intensity of the signal and spatial resolution. Similar to FT-IR, Raman spectroscopy probes different vibrational modes in molecules, measuring the shift in frequency of scattered light. This scattering pattern is unique and indicative of chemical composition and structure and can thus be used to classify material composition when the spectrum is compared to a reference database. It has been predominantly used for microplastic analysis when coupled to an optical microscope, e.g., Raman microspectroscopy (micro-Raman), and resolution can be further improved with the spatial filtering of a confocal optical microscope (confocal-Raman).

The benefit of the coupling with optical microscopy techniques is that it allows for further data on particle characteristics, such as physical size and morphology, to be collected. Classification of unknown or sample spectra involves comparing a particulate's spectrum with that obtained from a reference material. To enable these comparisons, researchers often use commercial spectral libraries such as SLOPP, BioRad's KnowItAll, and Omnic Picta Polymer Libraries.^31,33,37,40,42,44,72,73^ These libraries provide a wide range of reference spectra for various polymeric materials and thus play a critical role in identifying and classifying microplastics in different samples. Researchers also develop in-house libraries tailored to their specific research needs.^34,69^ These chemometric analyses are instrumental in confirming microplastic presence in samples.

Spectral acquisition is usually performed either manually, on a particle-by-particle basis,^37^ or semi-automated, where computational tools are utilized. With the aid of a motorized stage, both FT-IR and Raman can be used in imaging modes, where spectra for every pixel in an image/defined sample area are automatically acquired, or they can be used with particle-finding software, which detects the presence of particulates on a substrate using computer vision algorithms. This improves efficiency by prohibiting the acquisition of background spectra, focusing on particle targets.

Alternative techniques for microplastic analysis include pyrolysis-based techniques (pyrolysis gas chromatography–mass spectrometry, or py-GC-MS, and thermal extraction desorption-GC-MS). These techniques thermally degrade the plastic polymers, and the characteristic breakdown products (markers) detected in the GC-MS are used to qualify and quantify the mass content of plastic. While these methods are robust and higher throughput and quantify the amount of plastic, they do not generate any data concerning the size or shape distribution of the microplastic particles. Additionally, for samples such as tissues, having extra spectral imaging data evidencing microplastic particles embedded in tissue in situ would increase the reliability of findings.

This review aims to present an up-to-date overview of the application of vibrational spectroscopy-based microplastic measurements for understanding human exposure. Using the key concepts of “microplastic”, “exposure”, and “microspectroscopy”, search strings were constructed and combined with the Boolean operator “AND” (Table I) before searching in PubMed (11 April 2023). Filters were used to restrict results to the last five years, to ensure the literature is up-to-date, and to full-text availability. Finally, upon title and abstract screening, articles were excluded if they were on environmental monitoring or presence in biota from an ecological perspective; and if only the gastrointestinal tract and gills of commercial fish species were investigated, as these tissues are deemed less relevant to human exposure. Articles were included if they were deemed relevant to human exposure, such as observational studies focusing on air, dust, drinking water, and food, or experimental studies focusing on a direct point source release, e.g., from packaging. This reduced 1073 articles to 52, forming the bulk of this review, with hand-searched articles contributing to the introduction and discussion points. Laboratory-based methods are considered, assuming sample collection has occurred.

**Table I.** Search strings used to retrieve literature.

((((microplastic*) OR (nanoplastic*)) OR (microfib*)) OR (polymer))
(((exposure) OR (“human exposure”)) OR (“exposure assessment”))
((((((((Raman) OR (FT-IR)) OR (microspectroscopy)) OR (spectroscopy)) OR (“Fourier transform infrared spectroscopy”)) OR (“correlated imaging”)) OR (“SEM-Raman”)) OR (“nano-IR”))

## Summary of Articles

**Figure 1.**
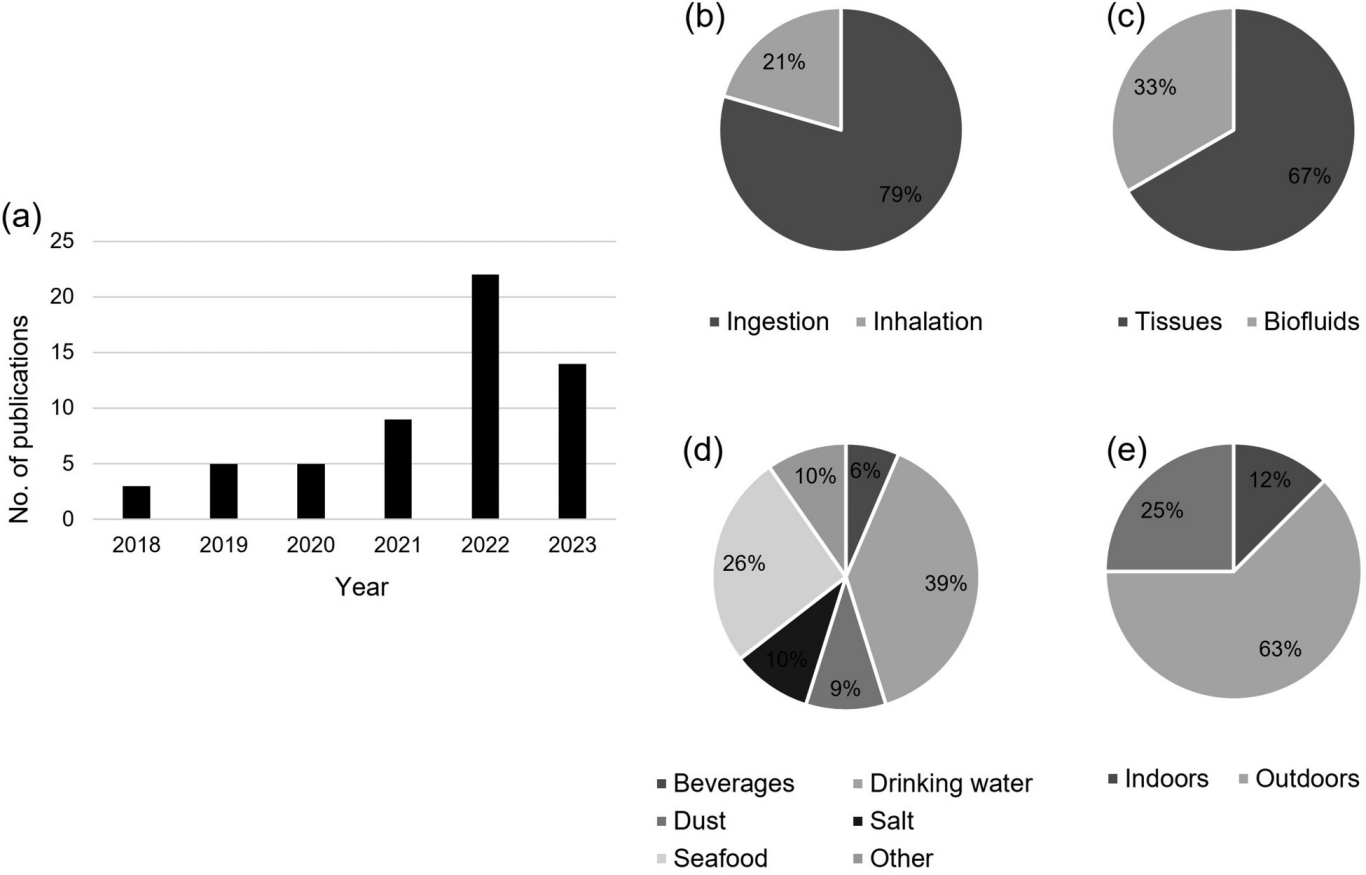
Summary of the reviewed articles. (a) The number of publications over time for the last five years; the percent distribution of publications by (b) external exposure pathway, (c) internal exposure category, (d) oral exposure category, and (e) inhalation exposure category.

In total, 52 articles were considered appropriate for this review (Table S1, Supplemental Material). The publication rate of articles on this topic is increasing (Figure 1a), emphasizing that this is a growing field. Seventy-five percent of the articles focused on an external exposure pathway (oral or inhalation), 23% focused on internal exposure (tissues/biofluids), and one study (2%) on deposition in the surgical environment^17^ was categorized as “other”. This study is included as it could be important to the interpretation of results on internal exposure. Of the studies on external exposure, 79% were on matrices relevant to ingestion, whilst 21% were relevant to exposure via inhalation (Figure 1b). For internal exposure, 67% of studies were on tissues, while 33% were on biofluids (Figure 1c).

With respect to oral exposure, the majority (39%) of studies were on microplastic contamination of drinking water, followed by seafood (26%). Other beverages, salt, and dust were also studied, and those matrices with only one study were categorized as “other” (breast milk storage bags, orthodontic aligners, sugar) (Figure 1d). For drinking water specifically, matrix categories included bottled (50%), ground (8%), kettle (8%), drinking water fountain (“kiosk” 17%), and mix (17%). Of the studies focused on air and thus the inhalation exposure pathway, most (63%) were on outdoor air, 12% were on indoor air, and 25% analyzed both outdoor and indoor air (Figure 1e).

## Sample Preparation and Processing

These studies adopted a range of sample preparation and analytical techniques, which are detailed below.

### External Exposure

#### Drinking Water and Beverages

The most common sample preparation step taken for liquid-based samples is filtration. This is because the liquid samples tend to be a simple matrix, with low viscosity leaving behind little residue and thus presenting minimal signal interference during spectroscopic analysis. Filtration has been used to extract microplastic from groundwater,^53^ water distribution pipes,^54^ tap water,^74^ drinking water fountains,^58,59^ bottled water,^29,52,55–57,74^ soft drinks,^23^ steeped tea bags,^75^ and breast milk storage bags.^76^ The sample is either directly analyzed on the filter or extracted and prepared as a drop-cast on a more suitable substrate. Anodisc (alumina oxide) membrane filters have been used for FT-IR-based analyses,^53,54,74^ as have cellulose,^56,58^ PTFE,^57^ and glass fiber filters.^52^ It is likely that particles were manually extracted and transferred to the instrument from the latter filter substrates, as ATR FT-IR was performed, avoiding interference from the active filter background.

Alternatively, following varying degrees of processing, drop-casts may be prepared on slides. Microplastic filtered from bottled water was extracted from a stainless-steel membrane into ethanol (EtOH) using sonication. The resulting suspension was concentrated down under nitrogen, and then the entire volume was prepared as a drop cast on a reflective glass slide.^55^ This is similar to a study that qualified the composition of concentrated particles extracted from tea bags on aluminum foil,^75^ and another study that prepared drop casts from atmospheric particulate samples for micro-Raman.^30^ Centrifugal filtration was used to concentrate micro- and nanoplastic particles released from the mechanical wear of PET bottles following opening and closing cycles. One microliter of the resulting solution was drop-cast onto a silicon wafer for subsequent Raman analysis.^77^ The simplest sample preparation is a direct drop cast of an aqueous suspension, without extraction/filtration, as performed for irradiated plastic bottled water samples, which were prepared on a silicon wafer.^78^

In cases where there is a level of organic matter likely to interfere with spectral classification, chemical digestion might be necessary to purify the sample and concentrate the target. For water samples from outdoor refill kiosks (drinking water fountains), Fenton's reagent [30% H_2_O_2_, 0.05 Fe(II)] was used, heating the samples to 75 °C for 1 h, before filtering onto mixed cellulose ester filters (0.22 µm).^58^ Heating samples above 50 °C is not advised prior to microplastic analysis due to the glass transition temperature of some polymers (e.g., 60 °C for PA), which may cause them to agglomerate.^9,79^

#### Soluble Food: Salt, Sugar

Salt and sugar samples, obtained either from grocery stores or from factories, were primarily prepared by digesting any co-occurring organic matter through the addition of hydrogen peroxide (H_2_O_2;_ potassium peroxide, or KOH, 30% v/v). The digested solution is then filtered, and the resistant microplastic particles are captured on the filter, which is subsequently analyzed by microscopic observation and spectroscopic analysis. The sample is either dissolved in water before digestion or H_2_O_2_ is added directly to the solid sample without prior dissolution. Cellulose nitrate was the main filter substrate used, with varying pore sizes, from 0.45 µm to 0.8 µm to 5 µm. In some studies, the filter paper was dried at room temperature and in other cases, drying was not mentioned.^60,61,80,81^

#### Seafood: Bivalves, Crustaceans, and Fish

Microplastic contamination has been reported for a range of seafood species. For bivalves, varieties of mussels (*Mytilus galloprovincialis*, *Mytilus edulis*, *Mytilus* spp., *Modiulou modiolus*, and *Perna viridis*) and Scottish scallops (*Pecten maximus*) have been analyzed.^62–64,82^ For crustaceans, shrimp (*Plesionika naval*) have been analyzed.^83^ A range of fish species, e.g., icefish (*Neosalanx* spp.), Scottish haddock (*Melanogrammus aeglefinus*), Greek seabass (*Dicentrarchus labrax*), and Icelandic plaice (*Pleuronectes platessa*), have also been analyzed.^65,84^ In addition, processed fish such as canned tuna, canned salmon, and canned sardines have been analyzed.^85^ Sample sources include wild, aquaculture, or grocery stores. Samples were mostly stored frozen until analysis (−18 °C to −30 °C), although temperatures were not always specified. In one study, samples were preserved in 10% formalin solution.^83^

Seafood is a complex matrix for which a degree of sample preparation is needed to extract microplastic from biological tissues. The main method used to do this is digestion. An exception is by Bordbar et al.,^83^ who manually separated microplastic from tissue under a microscope. Reagents used for digestion included the Carolase 7089 enzyme mixture, 10% KOH, 30% H_2_O_2_, and 30% H_2_O_2_ combined with the Fe(II) catalyst. Carolase 7089 was used for the mussels *M.* spp. and *M. modiolus* at 9.6 UHb/mL and 19.3 UHb/mL, respectively, leaving the tissue in the reagent overnight at 60 °C.^62^ For KOH digestions, temperature of 40 °C was used overnight with constant stirring (icefish)^84^ and for 72 h without stirring (mixed fish spp.).^85^ Bošković et al.^82^ left mussels in KOH at room temperature for three weeks without stirring. For all studies using H_2_O_2_, 60 °C or 65 °C was used, with stirring for 24 h.^63–65^

After digestion, the digestate is filtered, capturing the resistant microplastic particles. The filter substrates used include cellulose, cellulose ester, cellulose nitrate, and glass fiber. The pore sizes of the filters were 0.8 µm, 1.2 µm, 5 µm and 8 µm.

#### Dust

The preparation of dust samples for microplastic analysis has included wet peroxide digestion (50 mL 30% H_2_O_2_ 48 h) to remove the organic content,^26^ and density separations, in saturated sodium chloride (NaCl^26^ or zinc chloride (ZnCl_2_),^27^ to partition lighter plastic from heavier particles, such as minerals).^26^ In one study,^28^ no preparation was performed, and dust was collected from settlement plates by rinsing the surface with Milli-Q H_2_O. The resulting particle suspensions are then vacuum filtered, such as on mixed cellulose ester (0.45 µm)^26^ or glass fiber filters (1.2 µm^27^ and 0.6 µm^28^).

#### Air: Indoors, Outdoors, and Personal Exposure

A variety of samplers are used for sampling airborne microplastic, which can be divided into two methods: one is to actively draw air through a filter, onto an impaction substrate, or into an Eppendorf vial or liquid using a pump, and the other is to passively collect microplastic onto a surface as they settle naturally from air and then wash and collect the particles from the surface and process in the laboratory. Examples of indoor environments, which have been studied include residential, office rooms, university laboratories, public libraries, and foyers.^66,86–88^ Examples of the filters used for indoor air sampling include silver membrane filters (1.2 µm pore size), Teflon filters (0.2 µm pore size), glass fiber filters (1.6 µm), and a stainless-steel mesh screen (1 µm). On the other hand, for outdoor air sampling, Teflon (0.2 μm pore size), glass fiber (1.6 µm and <1 µm pore size), cellulose ester (0.8 µm pore size), and a stainless-steel mesh screen (1 µm mesh size) have been used. Samples have also been actively impinged into deionized water. Air was drawn through a succession of interconnected reservoirs, with particles cascading out of suspension and impacting the liquid surface where they are trapped.^86^

Depending on the filter substrate chosen for air sampling, it may or may not be compatible with the planned analytical technique. For example, some filter compositions are routinely used in air quality monitoring, e.g., polytetrafluoroethylene (PTFE)/Teflon, but are incompatible with FT-IR or Raman due to background signal interference of the active substrate. Wright et al.^89^ compared a variety of filters to identify an optimum substrate composition for Raman spectral imaging. These included quartz microfiber filters (1.2 µm), PTFE (2.0 µm), mixed cellulose ester membrane filters (0.8 µm), alumina-based membrane filters (0.2 µm), and silver membrane filters (1.2 µm). The greatest intensities for microplastic particles were observed against the silver membrane filter.

It thus may be appropriate to extract a sample from the filter and prepare it on a suitable substrate for analysis. Levermore et al.^30^ extracted atmospheric particles from a PTFE filter by submerging the filter in 5 mL of filtered EtOH, followed by 5 min of agitation in a sonicating bath (40 Hz). The extracted particulate matter (PM) was dried, weighed, and resuspended in EtOH. A 100 µL aliquot was dried dropwise onto a foil-covered slide. Rahman et al.^87^ extracted collected particles from Teflon (PTFE) and silver membrane filters by sonicating the filters in methanol (MeOH). The extract was dried under nitrogen and resuspended in 40 µL MilliQ water. The samples were further sonicated and then a 5 µL drop-cast was prepared on a CaF_2_ slide. One study extracted particles from a silver membrane because it could not be directly scanned, i.e., using focal plane array (FPA) FT-IR.^66^ However, the same issue was not encountered by Wright et al.,^89^ who found the membranes remained flat after sampling and were able to be Raman imaged. Thus, the type of sampling device or local environmental conditions may be influential. However, silver membrane filters are not optimum for visual/manual analysis due to the textured background affecting particle contrast under bright-field imaging. Thus, they are only recommended for imaging applications.

In cases where direct analysis of the filter has been performed, some studies first stain the particles on the filter with Nile red,^67^ which fluoresces in nonpolar environments when irradiated with ultraviolet A light. For liquid-impinged samples, Xie et al.^86^ treated the samples with dilute hydrochloric acid (pH 3, 24 h) to remove calcium carbonate, which may interfere with downstream Raman spectroscopy measurements. The digested samples were then filtered through an alumina membrane (0.22 µm) for analysis. Azari et al.^90^ have recently reviewed sampling and sample preparation methods for airborne micro- and nanoplastic in depth.

#### Internal Exposure

Current studies investigating the presence of microplastics in various human biological matrices have interrogated sputum, breast milk, sperm, bronchial alveolar lavage fluid (BALF), placenta, colon, and lung tissue samples. Identifying microplastics in these complex biological matrices is challenging. To simplify the matrix complexity and concentrate the microplastic targets, samples are preprocessed before analysis. Preprocessing typically involves a chemical or enzymatic digestion to remove the organic matter, followed by a density separation to separate plastic from denser materials.

Of the biological tissue samples interrogated for microplastic presence, only placental samples were dissected before further processing. These samples were dissected using a metal knife and scissors. The subsamples were either cut to a specific size (1 cm × 1 cm × 1 cm),^34^ or cut from specific anatomical regions, e.g., maternal, fetal, or chorioamniotic membrane.^33,73^

Among the various methods of chemical digestion used in studies, KOH was the most frequent, used for placenta, colon, thrombi, breast milk, and enclosed body fluids at concentrations of 10% or 30%.^31,33,37,42,72,73^ H_2_O_2_ (30%) and nitric acid (HNO_3_, 68%) have also been utilized, for lung/veinous tissue and sputum/BALF, respectively.^44,69,70^ Additionally, enzymatic digestion has been employed in a study analyzing testis and semen.^71^ The ratio of sample to digestion reagent volume ranged from 1:10 to 1:32 (w/v). Incubation periods and temperatures for KOH digestions varied, from 60 °C for 7–10 h to 40 °C for 48–72 h being reported.^31,37^ Zhu et al.^33^ reported the use of oscillation, with samples being agitated at 120 rpm. Qiu et al.^70^ used an HNO_3_ digestion, incubating samples for 48 h at room temperature. Samples undergoing H_2_O_2_ digestion were maintained at 65 °C for 168 h, rotating at 85 rpm, until no visible tissue remained.^44^ Zhao et al.^71^ employed enzymatic digestion, incubating testes samples in an enzyme liquid system at 60 °C for 8 h, followed by incubation at room temperature for a 72 h period. A range of filter substrates have been used for concentrating the digested sample, from glass fiber filters,^42,72^ aluminum oxide,^44^ cellulose^37^ to stainless steel,^33,70^ and silver.^69^

One study conducted further particulate separation via a density separation. Huang et al.^69^ employed a zinc chloride (ZnCl_2_) density separation method with a density of 1.7–1.8 kg/L. HNO_3_–sputum digestate was added to ZnCl_2_, stirred for 2 min, and allowed to stand for 12 h.

### Notes on Filtration

Since most studies concentrate a sample on a filter prior to analysis, it is worth discussing issues with filter choice. As mentioned above, the choice of filter substrate is important if direct analysis of the sample on the filter is to be performed, to avoid signal interference. Additionally, the pore size choice is important, as this dictates the particle size limit and any reagents used (including solvents for cleaning) should be filtered to this. Platinum-etched polycarbonate (PC) membranes (0.8 µm) were used to extract microplastic from breast milk storage bags for downstream micro-Raman analysis.^76^ This is likely an optimum type of substrate due to the flat and reflective surface enhancing the Raman scattering, similar to using gold-coated PC membranes.^29^

If the intention of the analysis is to derive counts leading to particle number concentrations, then quartz or glass fiber filters are not appropriate since these are depth filters, with particles embedded within the matrix. Counting only those particles on the surface would lead to an underestimate. However, quartz and glass fiber filters shed fibers upon extraction and are thus still not appropriate.

## Quality Analysis/Quality Control (QA/QC)

A critical element of microplastic analysis is contamination prevention, since microplastic pollution is ubiquitous in most laboratory environments. Several measures, ranging in effectiveness, have been taken for the included studies, reported variably. These include a combination of working in a laminar flow hood, working in a closed fume hood, wearing nitrile gloves, wearing a cotton laboratory coat, washing equipment with solvent, covering with foil, reducing sample exposure, keeping samples covered, and avoiding plastic materials. The laboratory bench and work surfaces can be continuously cleaned with alcohol and/or water.^31,61,81–84^ In some cases, face masks have been worn as an extra precaution^58^ and a muffle furnace has been used to pre-clean glassware prior to use.^54,58^ Heating glassware to temperatures exceeding plastic polymers' thermostability (e.g., >550 °C) will result in any contaminating particles burning off as a removal process. A clean room, with a low concentration of airborne particulates due to air entering the room being filtered, is considered optimum for robust microplastic work.^34,74^ Otherwise, a laminar flow hood or equivalent is also considered good practice for minimizing background contamination.^54,56–58,67,76,81,82^

All reagents, including deionized water for equipment rinsing, should be filtered through filters that have the same or smaller pore sizes as the filters used for sample preparation and final analysis (e.g., Li et al.,^63^ Akoueson et al.,^65^ and Thiele et al.^80^). However, there is currently no standardization regarding the materials used for prefiltration. Some studies have reported the use of 0.22 µm syringe filters,^73^ 0.45 µm silver member or PTFE membrane filters (e.g., Huang et al.^69^ and Zhao et al.^71^), and 1.6 µm pore-size glass fiber filters (Whatman GF/A; e.g., Zhu et al.^31^).

The adoption of a plastic-free protocol is also common in microplastic sample preparation. This involves restricting the use of plastic materials in sample collection, processing, storage, pretreatment, and analysis, instead opting for nonplastic alternatives, such as glass beakers, vials, petri dishes, metal lids, aluminum foil covers, and stainless steel forceps.^31,33,71,73^ During sample processing, while the operator is not directly interacting with the sample, it is common practice to cover the samples with aluminum foil. This precautionary measure helps reduce the likelihood of contamination through atmospheric deposition.^34,68^

In the modern laboratory, there is a range of microplastic sources, from people and clothing to dust and air to plastic consumables. For accurate interpretation of results, it is important to monitor for background contamination. Environmental and procedural blanks are often used to do this. Environmental blanks are in situ blanks, positioned near the processed samples, intended to monitor the contribution of background contamination from the laboratory atmosphere during analysis.^31,71^ Procedural blanks undergo the whole process, but do not contain a sample. Deionized water,^23,52,57,64^ ultrapure water,^29,76,77^ distilled water,^61^ or a blank without a matched matrix^54,60,63,65,82,84^ have been used as procedural blanks. In some cases, the composition of the procedural blank is not specified.^62,66,67,74,88^ In other cases, individual reagents used in the protocol are analyzed.^27^ The results are then either subtracted from the raw sample data as a background or used to calculate the limit of detection (LOD) and limit of quantification. It is important to state how many blank replicates were included, and how the blank data was used. The more blanks that are included, the more reliable the determination of baselines and any corrections that are applied. Often, studies do not report the number of blank replicates included, or the findings of the blanks (e.g., Liu et al.^27^). Sometimes, the average total microplastic count of the blanks is deducted from sample data as a baseline (e.g., Perera et al.^68^), although it would be more accurate to do this polymer by polymer than by total count.

## Application of Techniques

**Figure 2.**
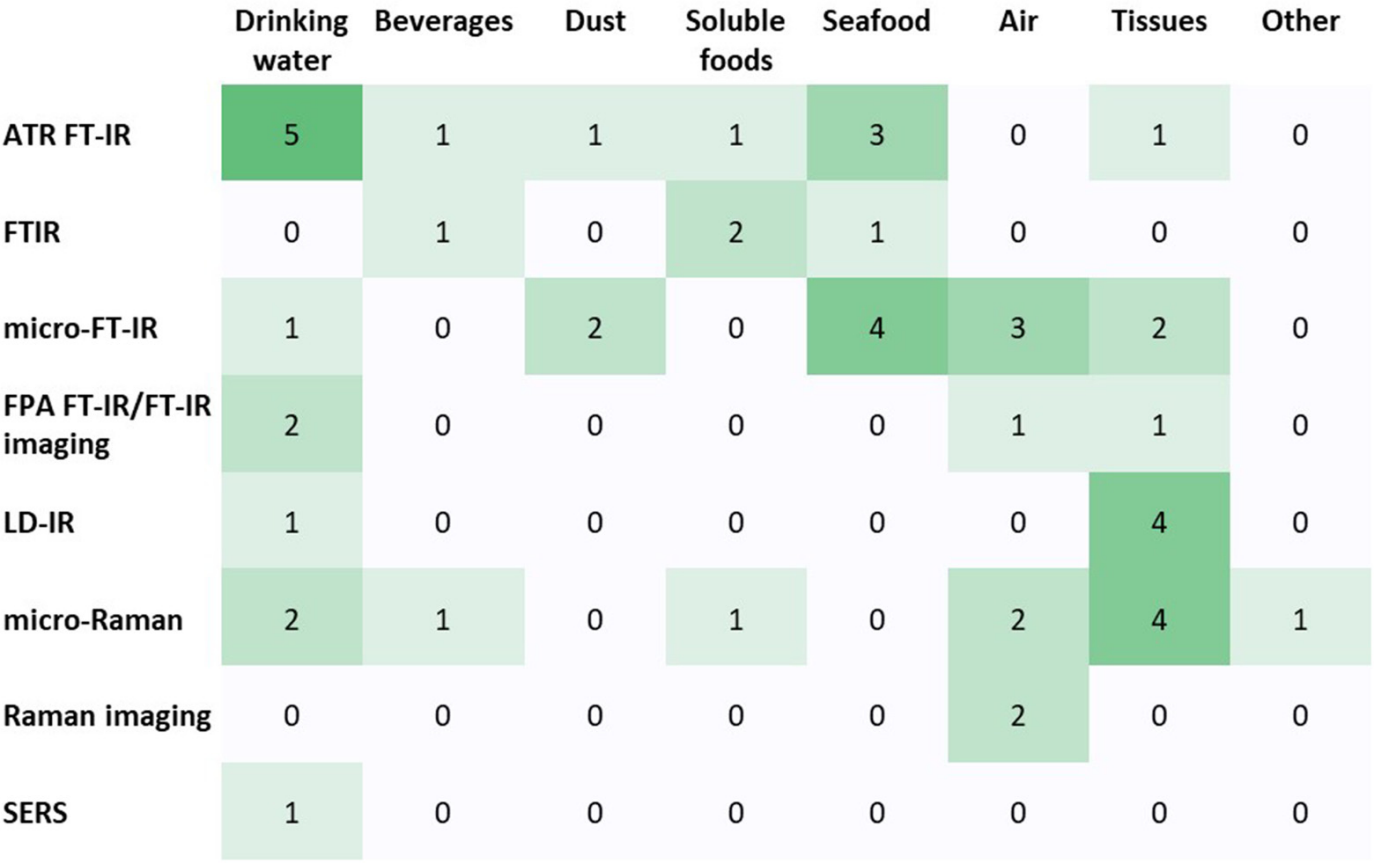
A heatmap of the distribution of spectroscopy techniques used for the different human exposure-relevant matrices. Numbers represent the number of studies which have used each technique.

Of all the included articles, 73% used a form of IR as the main vibrational spectroscopy mode for confirming the plastic identity of particles. The other technique used was Raman. Of the studies that used a variant of IR, the majority (37%) specified micro-FT-IR, followed by ATR FT-IR (29%). Other FT-IR techniques included FPA FT-IR (8%) and FT-IR (13%), which require the sample to be prepared as a compressed pellet. Studies using laser-direct IR (LD-IR) comprised 13% of the total. Most studies using Raman techniques used micro-Raman (79%), with 14% using Raman imaging, and one study (7%) using surface-enhanced Raman spectroscopy (SERS). How this breaks down by matrix type is shown in Figure 2.

## External Exposure

### Drinking Water and Beverages

#### Infrared

Of the studies on drinking water and other beverages, ∼67% used IR techniques to analyze samples. Most (60%) used ATR FT-IR,^52,56–59,75^ two studies used FPA FT-IR,^53,54^ one used FT-IR,^23^ one used micro-FT-IR,^74^ and one used LD-IR.^55^ Three studies did not specify the spectral range analyzed.^23,74,75^ For the studies that did, a range of spectral ranges were reported, largely spanning 400–4000 cm^−1^.^52,54,56–59^ Five studies had a spectral resolution of 4 cm^−1^ ^52,57–59,77^ and three had 8 cm^−1^,^53,54,56^ and the remaining studies did not report this. Spectral resolution is an important measurement parameter as the higher the resolution (i.e., the lower the wavenumber), the better the separation of similar IR bands in a spectrum, although at the expense of measurement time. Enhanced separation could be particularly useful for samples containing a range of unknown components, such as environmental and tissue samples. For microplastic analysis, a 4 cm^−1^ resolution is desirable as a higher resolution does not improve the data for solids and liquids. Some studies analyzed a focused spectral range. For example, Mintenig et al.^53^ took two FPA FT-IR images at 1480–1430 cm^−1^ and 1790–1700 cm^−1^, as polymer-specific regions, and then manually inspected the resulting particles highlighted in the image against a library search. Winkler et al.^77^ focused on 1300–4000 cm^−1^. The study using LD-IR used a 900–1800 cm^−1^ spectral range. Two studies specified using transmission mode.^54,77^

When classifying spectra using a reference library, a hit quality index (HQI) above 70% is generally accepted as good practice, with anything below potentially susceptible to bias and inaccuracies. Several studies used in-house libraries and manual inspection/cross-checking of the spectra.^74,75,77^ While this is appropriate for controlled studies on focused materials, it is likely challenging to classify environmental microplastic spectra in this way due to lower signal-to-noise ratios (S/N) and interfering bands. Most studies used a commercial spectral library to match unknown spectra. Two used an HQI of 70% or above^58,59^ and one used an HQI of 85% or above.^55^ One study accepted a match as 60% or above,^56^ and two studies specified 70% but not the use of a library.^23,52^ Taheri et al.^57^ classified sample spectra by visually matching them against published polymer spectra, which likely led to inaccuracies. In some cases, a library or software was used, but HQIs were not reported.^53,54^

Illustrating the application of different techniques, FT-IR was used to classify microplastic particles contaminating 10 brands of soft drinks in two cities in Turkey.^23^ On average, low microplastic concentrations were observed (mean 8.9 microplastic particles/L), however, a restricted size range was considered, limited to particles that could be manually transferred to a clean filter. Nevertheless, particles as small as 27 µm were counted. Whether this particle was confirmed to be of plastic composition or not using FT-IR is unclear and the type of FT-IR used was not specified. On average, particles measured 214 µm in size and were predominantly comprised of PE, PET, and PA.^23^ In a study from the same group on bottled water, a mean of 4.6 and 12.6 microplastic particles/L comprised of PE, PET, PP, and PA were found in commercial brands of plastic and glass bottled water, respectively, using ATR FT-IR.^52^

Attenuated total reflection Fourier transform infrared spectroscopy (ATR FT-IR) has also been applied to analyze bottled water samples from Thailand^56^ and Iran.^57^ Kankanige and Babel^56^ analyzed only those particles visible to the naked eye which were able to be manually transferred to the instrument with this technique. Those smaller ones were analyzed using micro-Raman (detailed below). Nile red staining was also used for particle counting (following spectroscopy measurements), although not all stained particles are necessarily microplastic. PET, PE, PP, and PA were classified using FT-IR, 384 of the 839 particles (>50 µm) included for analysis across 20 samples were identified, presumably as plastic although the authors do not say. Additionally, the authors do not specify how many particles were analyzed using ATR FT-IR, and how many were using micro-Raman. They also do not say if the reported results are corrected for the identification rate of plastic and use an HQI of 60% and above, which is below the recommended 70%. Thus, these data should be interpreted with caution. Taheri et al.^57^ analyzed 25% of particles per bottled water sample. Spectra were classified visually against in-house reference spectra. Despite analysis being performed directly on a PTFE filter, and hence the potential for background interference, the authors were able to attribute spectra to PET or PP as the main plastic types and the bottle as the main source of contamination. On average, 1496.7 ± 1452.2 microplastic particles/L were quantified in bottled water, and >90% were <10 µm, although this is likely below the LOD of the micro-FT-IR and thus may not have been confirmed as plastic in composition. Freeze–thawing did not impact the amount of microplastic released from the bottles, but mechanical stress and exposure to sunlight significantly increased the amount of microplastic released. However, all bottles prior to sunlight exposure had no microplastic in the water, and this contradicts the other findings reported for microplastic in bottled water without sunlight exposure and prior to stress tests. Along with the potentially biased identification method, these findings should be interpreted with caution.

Similarly, micro-FT-IR ATR (LOD 20 µm) has been used to classify the spectra of suspected microplastic particles isolated from public drinking water fountain (kiosk) samples in two studies in Mexico.^58,59^ Perez-Guavera et al.^59^ found a mean of 42 microplastic particles/L across 63 drinking fountains, with the variance not reported. PET constituted 37%, whilst vinyl polymers, i.e., high-density PE, polyvinyl acetate (PVA), polyvinyl ester resin, and polyvinylidene chloride, comprised 26%, and polyamides formed 25%. Size was poorly reported but most particles seem to be between 20 and 100 µm (20 µm LOD). In a second study from the same group, an average of 74.18 ± 48.76 microplastic/L was quantified across 22 fountains, with PVA comprising the majority (26%). Most particles were again categorized as 20–100 µm (20 µm LOD).^58^ Human exposure and health assessment were discussed in both studies. Briefly, Perez-Guavera et al. assessed microplastic exposure by multiplying the average microplastic concentrations by the daily water consumption from public drinking water fountains. This led to an estimated annual consumption of 1.47 × 10^4^ microplastic particles per adult and 6.73 × 10^3^ microplastic particles per child.^59^ Under their Discussion section, Human Microplastic Exposure and Health Assessment, Shruti et al.^58^ performed the same exposure assessment and discussed this in the context of potential adverse effects although without estimating the risk or impact on health.

In a different type of study, ATR FT-IR was used to confirm the composition of micro- and nanoparticles released from steeped synthetic tea bags.^75^ Particle number concentrations were derived from scanning electron microscopy (SEM) images of dried aliquots, and the chemical composition was confirmed to be nylon or PET, matching the original tea bags. The release of 11.6 billion microplastic and 3.1 billion nanoplastic particles per cup of tea was estimated. However, the chemical composition of a concentrated monolayer, rather than of individual particles, was acquired. Thus, whether all counted particles were plastic and not, e.g., precipitated oligomers, is unknown.^75^

Laser-direct infrared (LD-IR) has been used to classify and count the number of microplastic particles in bottled drinking water in China. In transflection mode, 17 different polymers were detected and classified using commercial and in-house spectral libraries. PVC comprised the vast majority of microplastics detected using this method, in both PET plastic and glass bottles. The methodological LOD was 10 µm, since this was the pore size of the filter used to extract the sample. Most particles in bottled water and tap water were in the 10–50 µm size fraction (67.85% and 75.50%, respectively).^55^

Fourier transform infrared spectroscopy (FT-IR) imaging has been applied to quantify the number and classify microplastic particles in raw water and drinking water samples in Germany. Fourteen out of 24 samples did not contain any microplastic (50–150 µm), whilst five samples contained <1 microplastic particle/m^3^ of water. One sample contained seven microplastic particles/m^3^ of water. Using FPA FT-IR, polyester (PES) was the most common polymer found (62%), although this was mainly influenced by two samples. PVC (14%), PA (9%), epoxy resin (9%), and PE (6%) were also found along the purification and supply chain.^53^ The LOD was not reported. Using FPA FT-IR imaging, microplastic particles were quantified in samples across the water distribution chain in Sweden (at a pumping station and corresponding hydrants for two pipelines). Twenty images were acquired for a subsample (20–52%) of each sample and corresponding procedural blanks, with 9.4 million spectra generated per scan. Across all samples, an average of 174 ± 405 microplastic particles/m^3^ were detected, ranging from 0 to 1219 microplastic particles/m^3^ in one sample from a pumping station. The samples were found to be dominated by PES or PA and 32% were below 20 µm in size, with the smallest observed particle measuring 8 × 5.2 µm.^54^ While this study employed a robust analytical protocol, the sample preparation included multiple steps. Quite high levels of background microplastic contamination were observed (average 46 microplastic particles/blank sample, maximum 155 microplastic particles/blank sample), which could occur during the multiple sample preparation steps. This study used an additional analytical technique, py-GC-MS, to quantify the number of target plastics in the samples. Interestingly, the two techniques were contradictory, micro-FT-IR detected PE, whilst py-GC-MS did not. This could be due to PE being below the LOD for py-GC-MS. FT-IR imaging was also used to analyze filtered drinking water samples from different sources in Saudi Arabia, mainly plastic bottled water. Lower and upper bound mean microplastic (25–500 µm) concentrations of 1.9 ± 4.7 microplastic particles/L and 4.67 ± 13.0 microplastic particles/L (majority PET) were found, respectively.^74^ However, only two procedural blanks were run, thus the level of confidence in the background is weak, the volume of water processed per sample was not specified, and the methodological LOD was 25 µm, which is large compared to the sizes considered of importance for human exposure.

#### Raman

Most studies using Raman spectroscopy to analyze microplastic in drinking water and beverages used a laser wavelength of 532 nm,^29,76–78^ with one study using a 672 nm laser wavelength.^56^ One study specified laser power (30 µW)^78^ and one study specified the gratings used (600 mm).^76^ Three studies specified the Raman shift window, which included 1300–4000 cm^−1^,^77^ 200–3200 cm^−1^,^29^ and 0–6000 cm^−1^.^56^ For the classification of spectra, three of the four studies did this manually.^29,77,78^ Since these were all controlled experiments with a pure source of microplastic, this is likely appropriate. Liu et al.^76^ used a combination of commercial and in-house libraries, although did not specify the HQI used to classify spectra. Kankanige and Babel^56^ used an in-house library for Raman spectral classification (>60% HQI).

Most studies utilizing Raman spectroscopy were experimental, where micro-Raman was used to classify the composition of microplastic particles released from packaging and products, thus enabling emissions and exposures to be estimated. For example, the chemical composition of particles released from breast milk storage bags was confirmed to be plastic.^76^ Using a 532 nm laser and commercial and in-house spectral libraries, particles were classified as PE, PET, PA-6, or were nonclassifiable. The spectra that were nonclassifiable suffered strong fluorescence interference.

Additionally, micro-Raman (LOD 0.5 µm) was used to identify the chemical composition of particles released and concentrated from PET bottles following opening and closing cycles. However, it was concluded that the mechanical stress degraded the materials and hindered their identification. The spectra from particulates exhibited a marked loss in intensity, a high S/N, and broad spectral features, which were poorly defined compared to the bulk material.^77^ Raman was further used to explore the chemical nature of the particles, for which peaks within their spectra were classified as amorphous or trans, but not crystalline orthorhombic phase.

In a study investigating the role of boil cycles and ionic composition of water on the release of microplastic from a plastic kettle, micro-Raman (LOD 1 µm) was used to confirm the composition of released particles, concentrated on a gold-coated PC filter.^29^ The spectra were inspected manually, focusing on bands in the ranges of 2780–2980, 1400–1640, and 709–850 cm^−1^,^56^ however, little detail is given concerning how many particles per filter were inspected and analyzed, and how the concentration estimate was derived. Considering the high concentrations observed (up to 25 to 30 million microplastic particles/L), there is likely some error in the extrapolation of a presumably small number of Raman data points to the numbers of particles observed. However, boiling water containing different ions over time reduced the number of particles released by up to 97% of the original release. Approximately 80% of the observed particles were <5 µm in size.^29^

Surface-enhanced Raman spectroscopy (SERS) has been applied to quantitatively investigate the experimental release of particles from plastic bottles and cups following irradiation. A 500 parts per million (ppm) concentration of CuO and Ag nanoparticles was combined with an equal volume of bottled water, drop cast, dried, and inspected with a 532 nm laser. This method had a trace LOD of 1.6 ng/L and found up to 3751 ± 0.19 ng/mL and 1522 ± 0.21 ng/mL of PE microplastic was released after 240 min of irradiation for plastic cups and bottles, respectively. Using complimentary SEM, an average particle size of 436.0 ± 15.5 nm was observed.^78^ This seems to be a promising method for microplastic extracts, although there could be potential for matrix interference from more complex sample types, and the exact composition of the SEM analyzed particles was not confirmed.

One study used confocal Raman to classify particles that were too small to be manually transferred to an ATR FT-IR (see FT-IR discussions above).^56^ Thirty-eight of 100 particles (6.5–50 µm) across samples were found to be plastic by confocal Raman, predominantly PE, PP, and PET. Across all bottled water brands analyzed, an average of 81.0 ± 3.0, 26.0 ± 2.0, and 12.0 ± 1.0 microplastic particles/L for 6.5–20 μm, 20–50 μm, and ≥50 μm sizes, respectively, were calculated. However, only five “spots” per sample were analyzed using confocal Raman. No indication of the percent of suspected microplastic analyzed per sample is reported and no information on how sample spectra were classified is given. Thus, the utility of the data is uncertain.

#### Soluble Food: Salt and Sugar

ATR FT-IR,^60^ FT-IR,^61^ and micro-Raman^80^ were used for identifying microplastic in salt, while FT-IR was used for sugar samples.^81^ For both salt and sugar analyses, the FT-IR spectral range analyzed was from 700 to 4000 cm^−1^. For Raman analysis, a 785 nm laser was chosen to limit fluorescence. The spectra were compared with a spectral library database to identify the chemical composition of the particle. Unknown spectra were matched using either an HQI > 80%, or with additional visual assessment if >70% but <80%.^60,80^ Afrin et al.^81^ adopted an HQI of 60%, which is below the recommended 70%. Ujjaman et al.^61^ reported using a library, but not an HQI, and mentioned that the acquired spectra were “contrasted to absorption bands of polymers reported in the previous studies”.

Following preparation, any solids including microplastic retained on the sample filter, were visually observed under a microscope and potential microplastic particles were identified by having homogeneous colour and an absence of cellular structures. The suspected microplastic particles were subsequently analyzed and classified by polymer type. Sathaish et al.^60^ and Ujjaman et al.^61^ also performed a hot needle test, i.e., pressing a hot needle against a particle (if it shows plasticity, it is likely plastic) to enhance the certainty that the particles were plastic. However, this implies the particles analyzed were quite large.

Using ATR FT-IR, the mean abundance of microplastic in unprocessed sea salts were reported as 35 ± 15 to 72 ± 40 microplastic particles kg^−1^.^60^ In unprocessed bore-well salt this was 2 ± 1 to 29 ± 11 microplastic particles kg^−1^.^60^ Through pellet-based FT-IR analysis, 195 ± 56 microplastic particles kg^−1^ were found in processed sea salt,^61^ while 343.7 ± 32.08 microplastic particles kg^−1^ were found in processed sugar.^81^ The one study using micro-Raman found 74 ± 105 to 1155 ± 140 microplastic particles kg^−1^ in European sea salts.^80^ The methodological LOD was 5 µm, due to the pore size of the filters (cellulose nitrate) used.

In all four studies, fibres were the dominant shape, followed by fragments, with spheres being the least common or not observed. Over 75% of microplastic particles in salt samples were fibres, whilst fibres comprised ∼38% in sugar (fragments and films were 28% and 25%, respectively). The size classification of particles varied between studies. The size bin <500 µm was most common for Sathaish et al.^60^ and Ujjaman et al.^61^ Thiele et al.^80^ found most fibres to be <155 µm in length, with a similar median diameter of 17.3 µm ± 8.0 and 16.9 µm ± 6.1 for Atlantic and Mediterranean salts, respectively. In branded and unbranded sugar samples, 60% of microplastic particles were below 300 µm.^81^ Whilst the sizes are still relatively coarse, in all four studies the majority of microplastic particles were in the smallest size bin. However, not all particles which were sized were also analyzed, thus there may be false positives and the size distribution is not specific to microplastic only.

In European sea salts, rayon, PP, PES, and PE were the main plastic types detected, but also nitrocellulose and copolymers (either ethylene/PS or acrylonitrile butadiene) were found.^80^ In sea and bore-well salts, PE, PP, PES, and PA were observed.^60^ From all processed and unprocessed sea salt, PET, PP, PE, were found.^61^ PS and nylon were also found but less frequently (PS was found in three processed sea salt samples out of five while nylon was found only in one branded sea salt).

#### Seafood: Bivalves, Crustaceans, Fish

All studies on seafood used a mode of IR spectroscopy as the analytical technique. Most (50%) used micro-FT-IR,^62–65^ almost 38% used ATR FT-IR,^82–84^ and one study (12.5%) did not specify the mode of FT-IR.^85^ Less than half the studies reported the spectral range analyzed. For those that did, these spanned 400 to 4000 cm^−1^.^62,84,85^ Less than half the studies reported the spectral resolution, however, when it was reported, it was always 4 cm^−1^.^62,63,84^ Two studies adopting micro-FT-IR specified using transmittance mode.^63,65^ Of the studies classifying spectra with a commercial library, 80% used an HQI > 70%,^63–65,84^ whilst one used a commercial library but did not specify the HQI used^62^ and one specified an HQI (>60%) but not specifically the use of a library.^82^

Of the studies using ATR FT-IR to analyze seafood samples for microplastic, average microplastic abundances of 2.52 ± 1.1 microplastic particles mussel^−1^ from the Boka Kotorska Bay of the Adriatic Sea were found.^82^ Fibres were the most common morphology and 5.6%, 26.2%, 18%, and 49.8% of particles were sized <0.1 mm, 0.1–0.5 mm, 0.5–1.0 mm, and 1.0–5.0 mm, respectively. In icefish (*Neosalanx* spp.), ATR FT-IR found an average 0.42 ± 0.28 microplastic particles g^−1^ of tissue (wet weight).^84^ PP, PE, PET, and PS were detected, and fibres were the most predominant particle shape (comprising 88.34%; *n* = 144). Particles were sized between 50 and 500 µm, with the 201–500 µm size bin being most common. ATR FT-IR was also applied to classify suspected microplastic particles isolated from the stomachs of commercial shrimp. Microplastic particles were detected in 5% (*n* = 146 out of 2411) of animals.^83^ Logistic regression found the number of microplastic particles in shrimp was related to month and geographical area. Microplastic particles were comprised of nylon (PA) by ATR FT-IR, without a dominant shape. Hussien et al.,^85^ using FT-IR, found tinned tuna to be contaminated with nylon, 1,2-polybutadiene, ethylene vinyl alcohol (EVOH), and sardines with EVOH, and poly(vinyl stearate), although did not specify concentrations.

Micro-FT-IR found 1.1 to 6.4 microplastic particles mussel^−1^ from coastal waters and supermarkets in the United Kingdom,^63^ to 7.32 ± 8.33 microplastic particles mussel^−1^ from wet markets in Thailand,^64^ and 3.4 ± 0.48 microplastic particles mussel^−1^ from Port Edgar.^62^ Ethylene/propylene copolymer, low-density PE, PP, PET, PES, poly (ether-urethane), PA and PVC were found. Fibres were the most common morphology of microplastic particles^62,63^ except for green mussels in Thailand, in which fragments were found to be the most common type.^64^ In the range of fish and shellfish species investigated with micro-FT-IR by Akoueson,^65^ a significantly higher number of particles were found in scallops, compared to fish. PET and PE plastic types were most detected, and fibres were the predominant shape throughout. Most particles in fish were sized 5–250 µm, whilst larger particles (500–5000 µm) were dominant in scallops.

## Other

One study focused on the potential for microplastic release from plastic orthodontic aligners. Micro-Raman (532 or 785 nm laser, 600 mm gratings) was used across a Raman shift window of 200–1800 cm^−1^, to confirm the composition (HQI > 80%) of particles released following wear simulation in water under constant stirring. Particles were found to be comprised of PET or PU, matching the original aligner material. Counts were performed in SEM images. Between 5 and 20 microplastic particles were released following seven days of simulated wear.^91^

### Dust

Just three studies analyzed dust for the contamination of microplastic. One study's primary analytical method was depolymerisation,^27^ micro-FT-IR was used as a supporting technique. One study used ATR FT-IR^26^ and one used micro-FT-IR.^28^ The spectral range analyzed was from 600 to 4000 cm^−1^. One study specified the spectral resolution (8 cm^−1^).^27^ All studies used a commercial library to classify sample spectra, however, whilst Liu et al.^27^ adopted an HQI of 70% of above, both Aslam et al. and Soltani et al. stated that where matches were <70%, manual inspection and comparison to the library was performed,^26,28^ which may be subject to biases or inaccuracies.

Attenuated total reflection (ATR FT-IR; 50 µm LOD) was applied to indoor house dust samples from across two cities in Lahore, Pakistan.^26^ Suspected microplastic particles were first visually discriminated and manually counted using a stereo microscope before a subsample of particles was analyzed. Fifteen fibres from all observed colour categories were analyzed. The number of particles (i.e., non-fibres) analyzed is not reported and it is unclear what proportion of total suspected microplastic particles were analyzed and how this was divided across samples. Additionally, whether the proportion of particles found to be microplastic was extrapolated to the total suspected microplastic count is unclear and no sizing is reported. Fibres were found to be the most common morphology, with 234.05 ± 148.41 m^−2^ (Lahore) and 159.02 ± 79.65/m^−2^ (Sahiwal) being calculated.^26^ PES, PET, ethylene–PP, PE, and PU were found. Particle sizes are not reported yet considered critical with respect to interpreting the potential to impact human health.

Micro-FT-IR (LOD 10 µm) was used to analyze suspected microplastic in pooled samples of outdoor and indoor dust from across China. Forty six percent of suspected microplastic fibres were found to be synthetic (PES, PU, nylon (PA), PE, PP and polyacrylonitrile), whilst 40% of suspected microplastic granules were confirmed as plastic (PES, PE, acrylic, PU, and alkyd resin). By extrapolating the proportion of successfully identified microplastic particles to a total particle count of suspected microplastic, it was estimated that adults and infants are exposed to 64.1 and 889 fibres kg^−1^ body weight d^−1^, respectively. Indoor dust was found to contribute to 97% of the total infant exposure via dust.^27^ However, only one replicate of each pooled sample was analyzed. The particles were manually selected for FT-IR analysis, it is not clear whether particles were directly analyzed on the filter or required manual transfer, which would restrict the particle size for analysis, although size data was not reported.^27^

In Australia, household dust was analyzed for microplastic using micro-FT-IR to extrapolate the proportion of true microplastic to a count of suspected microplastic. Out of 7401 counted fibres, which accounted for 99% of the counted suspected microplastic, 6% were analyzed to determine their composition. An average indoor deposition rate of 3095 synthetic fibres m^−2^ day^−1^ was calculated. However, the authors did not specify whether suspected microplastic were analyzed directly on the sample filter (glass fiber). A gold slide was used as a background, which is not appropriate if measurements were made on a filter. Additionally, where the library match was <70%, a vague description of how the spectra were matched is given.^28^ Given the emphasis on human health, including inhalation exposure assessment, this is not fit for purpose due to the large particle sizes analyzed (LOD 50 µm).

Interpreting the concentration of microplastic in dust is challenging. The studies are cross-sectional, i.e., representing a snapshot in time, and there is a range of human behaviors which can contribute to microplastic levels, which are not controlled for. Thus, how accurately the samples represent everyday exposure is unclear. For example, an inhabitant may be due to clean/hoover their home, or may have just cleaned/hoovered, which would influence the level of microplastic sampled and detected. Alternatively, one inhabitant may have their window open a lot, whilst another might not. Additionally, there may be more occupants in one household than another, effecting the level of exposure per individual. Aslam et al.^26^ try to account for this by having some controlled factors in the homes they sample from. A deposition rate, as acquired by Soltani et al.,^28^ is easier to interpret than a concentration of microplastic in dust or level for a given area.

## Air: Indoors, Outdoors, Personal Exposure

For analysis relevant to inhalation exposure, FPA FT-IR, micro-FT-IR, micro-Raman, and Raman imaging were used.

### Infrared Spectroscopy

Of the eight studies, three used IR techniques including FPA FT-IR in transmission mode^66^ and micro-FT-IR in reflection mode.^67,68^ One study used both micro-FT-IR and micro-Raman.^88^ The analyzed region was within 560 to 4000 cm^−1^, with one study not specifying this.^67^ The spectral resolution ranged from 2 to 8 cm^−1^.^66,68^ Amato-Lourenco et al.^67^ did not report this. Two studies used an HQI of >60%,^67,68^ and one study referred to using MPhunter software to classify spectra.^66^

Micro-FT-IR has been used to classify the composition of suspected microplastic particles in air samples. In Sri Lanka, outdoor and indoor airborne particulate samples were analysed.^68^ Micro-FT-IR was applied to random regions of the sample filters (stainless steel), analyzing 20 suspected microplastic particles per region or, if there were more than 20 suspected microplastic particles in a region, 50% of the particles were analyzed. Low concentrations were detected, ranging from 0.13 to 0.93 microplastic particles m^−3^ for indoor sites and 0.01 to 0.23 microplastic particles m^−3^ for nine out of eleven outdoor sites, with high variation between outdoor replicates (coefficient of variation 0–141%). Ninety eight percent of suspected microplastic were fibres in shape, with a median length of 551 µm (67 to 4919 µm). Indoors, 16% of suspected microplastic were confirmed as synthetic in composition, whilst outdoors 12% were. PET was the most common polymer detected. However, the authors report signal interference from the sample substrate (stainless steel mesh, 1 µm) and suggest that commercial reference libraries may not be capable of classifying the spectra of weathered microplastic particles. Whether the confirmed microplastic percent was applied to the total suspected microplastic count is unclear. Whilst the inclusion and summary of replicates contribute knowledge, particularly on the potential temporal variability in outdoor microplastic, the size of the particles counted and measured are large with respect to human exposure and would likely be swallowed and eliminated via the gut.

In a study investigating the presence of severe-acute respiratory syndrome coronavirus-2 (SARS-CoV-2) and microplastic in total suspended particulates in outdoor air surrounding the largest medical center in Latin America, PES was the most frequent polymer (80.4%) detected.^67^ Concentrations of total microplastic ranged from 0 to 24 particles m^−3^, depending on the sampling site, with significantly more fibres than fragments. However, only 5% of suspected microplastic particles were analyzed using micro-FT-IR. The extrapolation to the suspected microplastic particle count may thus be subject to error. Moreover, a HQI of 60% and above was employed for classifying composition; ≥70% is considered good practice. A significant positive association was found between microplastic levels and SARS-CoV-2 envelope gene quantities, however, not with nucleocapsid concentration, hence the findings are unclear.

To investigate the composition of particles sampled from outdoor and indoor air on a university campus, micro-ATR was used to analyze 122 particles. Ninety percent were found to be non-plastic. Of the 12 plastic particles identified, the majority were PS (46%), followed by PET (36.4%), with one particle each of PE and acrylic. A conservative HQI of 90% was used, which could account for the low numbers. Additionally, glass fiber filters were used for sample collection, which are not optimum since particles can embed in the depth. Hence, extraction via rinsing onto a slide, as performed, will not extract all the particles. No concentrations based on these findings are reported, likely due to the low numbers observed, although concentrations based on visual brightfield, and Nile-red-stained fluorescence counts are reported.^88^

Combined FPA with micro-FT-IR was applied as a new technique to investigate microplastic in air samples whilst avoiding the pre-selection of microplastic particles for analysis, thereby reducing data biases introduced by the analyst.^66^ Following an investigation on indoor (residential, office) air, a 24-h average concentration of 9.3 ± 5.8 microplastic particles m^−3^ was found. Of the total number of particles in the collected samples, microplastic comprised a small (4%) percentage. The most common polymer was PES (43%), followed by PA (22%), PS (17%), PE (13%), and PU (4%). With a LOD of 11 × 5 µm, the authors found a median size of 36 µm and 21 µm for the major and minor dimension, respectively, and this was slightly smaller than that for non-plastic particles (47 µm and 31 µm). The autonomy that this technique presents, in both data acquisition and analysis, is robust. However, the use of sonication to extract particles from filters should be performed with caution. Some studies have found sonication to fragment microplastic,^92^ potentially influencing particle count and size distribution data. Additionally, for human inhalation exposure assessment aligned to other environmental air pollution particles, the ability to detect smaller sized microplastic is needed. The adoption of a (aerodynamic) size selective sampling head could also aid this.

### Raman Specroscopy

Of the studies on samples relevant to inhalation and applying Raman spectroscopy, three used micro-Raman^86–88^ and two used Raman imaging.^30,89^ Three studies used a 785 nm laser wavelength^30,88,89^ and two used a 582 nm.^86,87^ Those using manual micro-Raman investigated Raman shift windows such as 100–3500 cm^−1^,^86^ 200–2000 cm^−1^,^88^ and 200–3200 cm^−1^.^30,89^ For studies employing Raman imaging, focused windows were used, centered at 1300 cm^−1^ (924.6 to 1668.0 cm^−1^ Raman shifts).^30,89^ Either 600 mm^30,89^ or 1200 mm^88^ gratings were used, or they were not reported.^86^ In terms of spectral classification, where commercial libraries were used, HQIs of 75%^86^ and 90%^88^ were adopted. Two studies used in-house libraries,^30,89^ one of which used software developed in-house for performing Gaussian cure peak fitting to identify plastic in an image based on their characteristic peaks,^89^ and one used Matlab to perform multivariate analyses to match and map spectra in Raman images to plastic spectra, using an HQI of 70 or 80%, depending on the plastic polymer.

In an aim to optimize a filter-based method compatible with both air quality monitoring and Raman microscopy and imaging, Wright et al.^89^ trialed a range of filter substrates, both clean and PM_10_-loaded, for the ability to detect model microplastic particles (PS_4,10 µm_, PE_10–27 µm_, PMMA_5–27 µm_) using Raman imaging and univariate analysis. The strongest plastic band intensities were acquired on silver membrane filters (quartz, PTFE, and cellulose were not appropriate), with model microplastic particles down to 4 µm still detectable after being environmentally conditioned in situ for 20 h with ambient PM_10_. However, there was some background interference from the PM sample for some of the plastic bands and the patterned surface of silver membrane reduced the optical contrast of particles and thus are not appropriate for manual/visual assessment or particle-finding software.

To advance understanding of human inhalation exposure to microplastic, a sample preparation and analytical method was further optimized for ambient PM, using Raman imaging and multivariate chemometrics.^30^ After trialing different slide substrates (gold-coated, aluminum foil-covered, stainless steel, low-e, CaF_2_), validating the method using positive and negative controls, and comparing supervised and unsupervised statistical methods, the optimized method was applied to an extracted outdoor PM_10_ sampled (24 h) from an urban traffic environment. Back calculation of the microplastic particle count in the Raman image analysis derived a concentration of >2500 microplastic particles m^−3^. Particles ranged from 4.7 to 40.9 μm, with 52% being 5–10 μm in size. Pearson's correlation coefficient was the statistical technique found to perform best, with the highest identification rate of microplastic particles spiked into an environmental PM matrix. The Raman imaging–chemometrics method was found to outperform manual, user-based microplastic identification, which failed to detect 94 ± 4% and 60 ± 20% of 2 and 4 µm PS microspheres, respectively.^30^

In a similar study, Rahman et al. also trialled the efficacy of different air sampling filters (PTFE, silver)/substrates (CaFl_2_, glass) for microplastic Raman analysis and extracted PM samples from filters into solvent (MeOH) for preparation on a Raman substrate.^87^ Potential particles were identified using particle-finding software. Whilst the analytical method detected 1 and 0.3 µm model PS particles against the filters, the particle concentrations were so high that monolayers formed. Classification of individual particles was not possible,^87^ thus the sizes are irrelevant. In environmental samples, nylon (PA) fibres, PP, PE, chlorinated PE, styrene copolymer, PVC, and PUR were detected. Most samples contained less than one microplastic per µg pf PM, although one sample had a concentration of 288 microplastic particles µg^−1^. Particle-finding software increased efficiency, from 1.5 h manual analytical time to 0.5 h.

Micro-Raman was applied to filtered particulates sampled from air directly into deionized water.^86^ Indoor and outdoor environments in Shanghai, China, were sampled. Nine regions per filter (∼15% of total sample) were analyzed, with suspected microplastic particles being identified in region images first. For 43 different classes (shape, colour, texture) of particles, at least three and mostly more than 10 particles were analyzed per region. Most spectra were dominated by pigments, and the software (KnowItAll) was able to deconvolute mixtures to reveal the polymer composition as well. Overall, concentrations ranged from 15.56 to 93.32 microplastic particles m^−3^, although no average is reported. In general, higher concentrations were observed indoors than out, except for one outdoor site. Fragments were dominant, over 20% of microplastic measured <10 µm in their longest dimension and most particles were PE (74%). Whilst a conservative approach to identification was taken, it is likely that many particles were overlooked due to the visually pre-selection of particles for analysis.

Gaston et al.^88^ used both micro-FT-IR and micro-Raman to investigate the composition of particles sampled from outdoor and indoor air on a university campus. Seventy-one particles (fibres and fragments) were analyzed, and 54% were found to be synthetic in origin. The most common synthetic particle is described as PVC-heat stabilizer (50%, *n* = 19), however, it is not clear whether this is a PVC particle containing heat stabilizer additive, or a pure heat stabilizer signal. Plastic additives were counted as microplastic, comprising 24% of synthetic particles, although whether the particles were truly plastic, or a non-plastic particle contaminated with additive is unknown. Actual plastic particles were rarely detected: PVC (5%, *n* = 2), PE (5%, *n* = 2), resin (5%, *n* = 2), acrylic (6%, *n* = 2), PC (3%, *n* = 1), and PS (3%, *n* = 1). As mentioned above (see FT-IR section), no concentrations based on extrapolation from these findings are reported.

## Internal Exposure

Investigations on microplastic levels in purified human tissue samples commonly utilize FT-IR (66.6%);^33,34,37,40,44,69–71^ and Raman (33.3%).^31,42,72,73^ One study combined py-GC-MS with LD-IR analysis.^33^ Below is a further description of instrument configurations used for the differing microscopy-based interrogations of biological samples for the presence of microplastic.

### Infrared

Of the studies on human tissue which employed IR analysis, the majority (50%) used LD-IR,^33,69–71^ 25% used micro-FT-IR,^40,44^ whilst one each used FT-IR^37^ and FT-IR-imaging.^34^ Six of the eight studies employing FT-IR reported the spectral range analyzed. These varied from 1250 to 4000 cm^−1^,^34,40,44^ 400 to 4000 cm^−1^,^37^ and 900 to 1800 cm^−1^ for the two studies using LD-IR. Just three studies reported spectral resolution, which ranged from 8 cm^−1^ to 16 cm^−1^.^34,40,44^ Three studies captured spectral activity in transmission mode,^34,40,44^ two in reflection,^37,70^ and two of the four studies using LD-IR reported using transflection, whilst one study using LD-IR did not report this.^69^ In terms of data acquisition, where a particulate exhibited spectra with an elevated background due to autofluorescence,^40^ three additional interrogations using micro-FT-IR were attempted. If these attempts proved unsuccessful in obtaining reliable spectra, the operator proceeded to another particle.^40^ One study pre-processed the collected spectra, which included spectral smoothing, baseline correction, spectral normalization, and data transformation.^44^

With respect to spectral classification, commercial^33,37,40,44,70^ and in-house libraries^34,69^ were used. The two studies using micro-FT-IR, employ a threshold (HQI) of above 70%.^40,44^ Braun et al.^34^ and Ibrahim et al.^37^ do not specify the HQI used. Of the studies using LD-IR, the thresholds for correct chemometric assignment varied, with values greater than 0.65,^69^ 0.8,^70^ or greater than 0.9^33,71^ being employed. The higher HQI, the higher the accuracy and therefore more robust the data. When analyzing digested testes samples, Zhao et al.^71^ first used an IR pre-scan at 1800 cm^−1^ to differentiate particles against a filter substrate (stainless steel, 10 µm). The applications are summarized below.

The one tissue type for which ATR FT-IR was used to analyze is colon. In digested colon tissue samples, an average of 28.1 ± 15.4 microplastic particles g^−1^ of colon was found. The mean size of the particles was 1.1 ± 0.3 mm.^37^ The majority of microplastic particles consisted of PC (90%), PA (50%), and PP (40%). The summed percent across these three polymers is 180%, so the actual distribution is unclear. Most of the observed particles were fibres (>96%). Based on this and the polymer distribution, one can assume the most common particle type was a PC fiber, however, there is limited information on the availability and application of PC fibres in industry. Additionally, given the average particle size (1.1 mm), with fibres ranging from 0.8 to 1.6 mm in length, it is difficult to comprehend that these particles were inside the columnar epithelial cells lining the colon or embedded within tissues. Without presentation of sample spectra against reference spectra, or reporting of the number of blanks samples analyzed, it is difficult to further interpret these findings.

Micro-FT-IR (LOD 3 µm) has been applied to both digested lung^40^ and digested veinous^44^ tissues by the same research group. Jenner et al. found microplastic particles in ∼85% of the tissue samples analyzed, with an average 0.69 ± 0.84–1.65 ± 0.88 particles g^−1^ of lung tissue when adjusted for background contamination.^40^ PP and PET were the most abundant polymer types found, representing 23% and 18%, respectively. The average particle length and width were 223.10 ± 436.16 and 22.21 ± 20.32 µm, respectively, with fibres accounting for 49% of the particles, followed by fragments (43%) and films (8%). We calculate that the smallest microplastic particle observed in this study had an aerodynamic equivalent diameter (D_a_) of 5.4 µm (following Henn^93^). All other particles observed had a D_a_ greater than this. D_a_ is an important property as it is proportional to the probability of deposition for a given anatomical region in the airway. Current ambient and occupational criteria suggest 50% of inhaled particles with a D_a_ of <10 µm and <4 µm penetrate the thoracic and alveolar regions, respectively. There is a small likelihood that the particle dimensions observed could have deposited in the lung. However, given that the instrumental LOD was 3 µm, it raises the question as to why a greater abundance of microplastic particles down to this limit were not observed as expected. What can be concluded is that if these particles truly originated from environmental exposure, most were in the airway, not in cells and interstitial tissue since they would be too large for active cellular uptake.

Rotchell et al.^44^ found that microplastic particles extracted from digested venous tissue had an average length of 119.59 ± 226.82 µm and width of 41.27 ± 62.80 µm. The methodological LOD was 5 µm. Alkyd resin was the most abundant plastic type (45%), followed by PVAc (20%), nylon–EVA (20%), PUR (10%), and PVAe (5%). On average, 14.99 ± 17.18 microplastic particles g^−1^ tissue was reported. However, the discovery of particles up to 1074 µm in size in the veinous tissue is surprising. Translocation rates of particles from the lung and gut mucosa to systemic circulation decrease as particle size increases. For instance, a systematic review and meta-analysis on particle translocation from the airway and alveolar lumen derived a particle size cut-off of 1 µm for translocation to the blood.^94^ This is supported by previous studies. For example, 40 µm latex microspheres were unable to penetrate the tracheal epithelium in guinea pigs following intratracheal instillation.^95^ Additionally, just 0.15% of 0.5 and 1 µm PS microspheres were found to be bioavailable, entering systemic circulation following application to the nasal epithelium of rats, compared to 0.3% and 0.6% for 0.1 and 0.02 µm microspheres, respectively.^96^ In the gut these size-based limits for different uptake mechanisms are proposed to be <150 µm for persorption, 1 µm for phagocytosis, <200 nm for endocytosis, and <10 µm for particulate uptake at the Peyer's Patches,^97^ with uptake rates through persorption being low (0.002%).^98^ The identification of microplastic particles in veinous tissue that are larger than these established size-based barrier and membrane restrictions challenges biological plausibility and requires further investigation.

Braun et al.^34^ conducted a study to examine the presence of microplastic particles (≥25 µm) in placenta, meconium, and stool samples using FT-IR-imaging. Digested placental samples contained PE, PP, and PU microparticles, while digested meconium samples contained PE and PP. Digested maternal stool samples contained PE (0.096 microplastic particles g^−1^) and PS (0.048 microplastic particles g^−1^), although so did the procedural blank, though the level is not reported, and this seems to be an *n* of 1. Therefore, caution is advised when interpreting the findings presented in this study. Additionally, the authors did not provide data on the shape and size of the identified microplastic particles and the level of microplastic in placenta and meconium samples was not reported. A major flaw in the study design is that only particles >50 µm were isolated and analyzed. As mentioned above, such particle sizes have an extremely low likelihood of crossing the airway or GI epithelium. The gut is the more likely barrier of the two due to the mechanism of persorption for particles up to 150 µm.

Laser-direct IR (LD-IR) microplastic analysis has been applied to sputum,^69^ BALF,^70^ testes,^71^ and placenta.^33^ In all four studies, the LOD was 20 µm. In sputum, the median microplastic abundance was 3.95 microplastic particles mL^−1^.^69^ In BALF, 0.2 to 141 microplastic particles g^−1^ of tissue were quantified.^70^ In digested semen and testes, 0.23 ± 0.45 microplastic particles mL^−1^ and 11.60 ± 15.52 microplastic particles g^−1^ were reported, respectively,^71^ whilst for digested placenta, 2.70 ± 2.65 microplastic particles g^−1^ was found.^33^

The distribution of polymer compositions varied in these different biological matrices. In sputum samples, 21 different plastic compositions were classified, with PU (33.95%), and PES (21.63%) being most abundant.^69^ In BALF samples, the predominant polymeric composition observed was PE at 86.1%, followed by PET (7.5%), PP (1.9%), PC (1.6%), and PU (1.4%).^70^ In testis samples, four polymer types were identified: PS (67.75%), PVC (12.9%), PE (12.9%), and PP (6.5%^71^;). In semen fluid, the microplastic abundance was observed to be 0.23 ± 0.45 microplastic particles mL^−1^,^71^ including PE and PVC. In placental tissue, PVC (1.19 ± 1.97 microplastic particles g^−1^; 43.27% of total microplastic) was the most abundant, followed by PP (14.55%), polybutadiene succinate (10.90%), PET (7.27%), PC (6.91%), PS (5.82%), PA (5.45%), PES fiber (2.91%), PE (1.45%), polyacrylamide (0.73%), and polysulfone (0.73%).^33^

All studies further categorized microplastic into particles and fibres. Fibres and fragments were most abundant in semen samples (29% each).^71^ The dominant morphology in testes samples were fragments^71^ as well as in placenta (∼67% of particles).^33^ The size range of microplastic particles varied, with placenta samples ranging from 20.34 to 307.29 µm and semen samples ranging from 21.76 to 286.71 µm (average 96.19 ± 74.17 μm).^71^ Zhao et al.^71^ and Zhu et al.^33^ report an increase in microplastic abundance as the size range decreased. In semen fluid and placental tissue, 67%^71^ and 80%^33^ of microplastic particles were sized 20–100 µm, respectively. In testes samples, 80.6% of microplastic particles were between 20–100 µm (average 83.15 ± 56.25 μm).^71^ Qiu et al.^70^ stated that “most MPs (in BALF) were within the size range of 20–80 μm”, but did not provide quantitative data on this. Similarly, Huang et al.^69^ describe most microplastic particles in sputum as less than 500 µm in size, with a median 75.43 µm (44.67–210.64 µm, interquartile range).

### Raman Spectroscopy

Human tissue samples analyzed for the presence of microplastic using Raman spectroscopy include breast milk,^31^ thrombi,^72^ placenta,^73^ and enclosed biological fluids.^42^ All studies used micro-Raman. The selected excitation wavelengths were either 532 nm^31,42^ or 785 nm.^42,72,73^ All studies collected broad spectral information, with slight adjustments to the Raman shift (cm^−1^) window. The Raman shift window ranged from either 200 to 3000 cm^−1^ or 200 to 3500 cm^−1^. Additionally, a grating with 600 lines/mm was consistently used across all three studies.^31,42,72^ Amereh et al.^73^ did not report microscope methodological parameters.

Spectral classification was predominantly performed using commercial libraries, with successful classification being set to >70%^72^ and >80%.^31,73^ Guan et al.^42^ did not report the threshold used for classification. Figures of classified spectra clearly demonstrate the impact of a 532 nm excitation source on classification. In the study by Guan et al., a PA-6 classification received an HQI score of 92.95.^42^ However, the resulting classification appears significantly attenuated to the overlapping signals in the C–H stretching zone, as there is limited similarity between the sample and reference spectra in the fingerprint region. This observation emphasizes how the choice of excitation source can affect the quality and distinguishability of spectral data, particularly in specific spectral regions, potentially impacting the accuracy of identification and results.

Among the studies investigating the presence of microplastic in biological matrices using Raman microscopy, one study expressed abundance in terms of microplastic number per gram of sample. Ragusa et al.,^31^ though not explicitly stating the average concentration of microplastic in their cohort of breast milk samples, calculated a mean abundance of 0.6 ± 0.7 microplastic particles g^−1^. The findings from Guan et al.^42^ are less clearly reported. They state that 1 to 19 microparticles 500 µL^−1^ of bodily fluid were detected, but also that the average particle number ranged from 29 to 80, with no units reported. Wu et al.^72^ identified one microplastic, low density PE, and 23 copper phthalocyanine particles in thrombi tissue. The authors do not report microplastic concentration (i.e., per gram of tissue), even though 1 g of thrombotic tissue was obtained from each participant.

In comparison to FT-IR, methods proposed for Raman microscopy had a smaller spatial LOD being >1 µm. Ragusa et al.^31^ observed that microplastic particles in breast milk were most abundant in the 4–9 µm size range (47%), followed by ≤3 µm (29%), and ≥10 µm (24%). Guan et al.^42^ found microplastic in enclosed body fluids to range from 19.66–103.27 µm in size, with an average of 49.52 ± 25.76 µm. Amereh et al.^73^ found the mean particle size in digested placental tissue to be 9.86 µm, ranging from 2.9 to 34.5 µm. In thrombi, the smallest particle observed was 2.1 μm. Sixty particles were smaller than 10.0 μm, six were larger than 20.0 μm and the largest sized particle was 26.0 μm.^72^ As outlined above, it is important to consider the plausibility of the biodistribution of microplastic particles of the sizes mentioned, especially since microplastic is not the only class of particles the population are exposed to in that size range, e.g., dietary starch, minerals, pollen, etc.

Classification of the spectra acquired for particles in digested breast milk samples revealed the most abundant polymers to be PE (38%), PVC (21%), and PP (17%).^31^ Forty-eight out of 58 samples were found to be plastic, whilst the remaining 10 spectra were dominated by pigments. In digested thrombi samples, 1% of observed particulates were identified to be microplastic; low-density PE (*n* = 1).^72^ Amereh et al.^73^ found PE was the predominant microplastic in placenta from both healthy (66.7%) and intrauterine growth restriction (43%) donors. PS represented 33% and 36.4% of the remaining microplastic in healthy and IUGR placentas, respectively. Two other polymer types, PET, and PP, were identified in IUGR placenta samples, comprising 14.9% and 5.6%, respectively.^73^

Guan et al.^42^ observed that the compositional distribution of plastics in enclosed body fluids varied. Utilizing the metadata available from the Supplementary Material, we calculated the relative proportions of microplastic polymers within each fluid compared to the total number of particulates, including non-plastics like graphite. The results revealed the following observations: (i) pericardial effusion the predominant polymers were PS (12.1%), polyvinyl butyral (6.0%), PP (3.0%), and polytetrafluoroethylene (3.0%); (ii) whole blood the LDPE was the most abundant polymer (3.7%), followed by PA-6 (1.89%), PS–acrylonitrile (1.89%), and PVA (1.89%); (iii) testicular effusion the most prevalent polymeric composition was PS at 4.44%, followed by PA-6 (2.2%) and PP (2.2%); (iv) pelvic cyst fluid PS accounted for 3.08% of the identified particulates; and (v) intrauterine effusion, two types of polymers were identified, namely PP (2.13%) and PS (2.13%). However, the HQI was not reported, hence results should be interpreted with caution as the accuracy of the classification is unclear.

The presence of, e.g., large, fibrous microplastic particles in semen and placental tissue, along with the presence of >100 µm particulates observed in other above mentioned biological samples challenges current biological theory on particle kinetics in the body. This raises a need for a mechanistic understanding of how particles of this size translocate into the studied sites of accumulation or excretion. Without rigorous mechanistic follow-up studies and more high-quality data from observational studies, it is difficult to verify the findings described.

## Fit for Purpose

Infrared and near-infrared spectroscopies have their own advantages and disadvantages. Interestingly, Gaston et al., when applying both micro-FT-IR and micro-Raman to filter-based indoor and outdoor air samples, found the results on polymer distribution were vastly different.^88^ In indoor air, PS dominated the FT-IR spectra, followed by PE and PET, whilst for Raman data from the same samples, PVC was prevalent, followed by PE. In outdoor air samples, there was an equal distribution of PS, acrylic, and PET in the FT-IR data, while Raman was dominated again by PVC. However, particles and fibres in a subset of samples were randomly selected for Raman analysis, and particles and fibres were then washed onto a slide for FT-IR analysis. There is potential that this difference is down to heterogeneity of polymer types on the filters, however, the commonality of PVC in Raman data for both outdoor and indoor samples suggest there could be a physico-chemical reason. Further work is needed to determine the comparability of IR and Raman microplastic data.

The choice of analytical technique will be guided by the research question and thus be a part of early study design. For microplastic exposure in humans, particle size is important. This is because, along with other properties such as surface coating and chemistry, it influences the trajectory of a particle in the body and therefore likelihood of uptake into tissues and internal dose. Accumulation potential is also important as current microplastic levels in air and in the diet are very low relative to the levels that have been found to cause adverse effects in laboratory studies. Thus, the likelihood of microplastic associated hazards will increase where microplastic particles accumulate if a threshold for toxicity is reached. A caveat is that the potential for adverse effects due to chronic exposure to low levels is unknown and difficult to model.

For ingested particles that are too large for cellular uptake and/or translocation at the gastrointestinal epithelium (i.e., >10 µm), gut transit and elimination via stool is predicted,^35^ as the rate of uptake for such particles up to 130 µm, through a passive mechanism called persorption, is low, i.e., 0.002%,^98^ meaning two particles for every 1000 ingested. Most studies calculated drinking water and beverage concentrations to be orders of magnitude lower than this. The likelihood of active particle (<10 µm) uptake across the gut increases with decreasing particle size and is also affected by surface charge. For example, the bioavailability (% in blood relative to original dose) of neutral and positively charged 50 nm PS nanoparticles was 0.3 and 0.2%, respectively, while for carboxyl-modified negatively charged PS_50 nm_, it was 1.5–1.7%.^99^ Of the 15 studies on microplastic in drinking water and beverages, six (five using FT-IR, one using Raman) do not specify the methodological particle size LOD. Of the nine that do, six are above 10 µm. FPA FT-IR analysis had a LOD of 6.6 µm,^54^ and micro-Raman had the most resolved LOD, ranging from 0.5 µm^77^ to 1 µm.^29,56^

For inhaled microplastic particles, aerodynamic diameter, a factor of material density, shape, and size is important, as it dictates the likelihood of where a particle will deposit in the airway. This influences air quality guidelines and monitoring; those particles with an aerodynamic diameter <10 µm (PM_10_) have an increased likelihood of depositing in the central and branching airways, whilst those with an aerodynamic diameter <2.5 µm (PM_2.5_) have an increased likelihood of depositing in the deep lung (alveolar region). These different regions have different clearance mechanisms which occur at different rates. Particles depositing in the central airways undergo mucociliary clearance on the order 24–48 h, whilst particles depositing in the alveolar region undergo macrophage clearance on the order of months. Decades of research has led to the conclusion that elevated exposures to PM_10_ and PM_2.5_, as opposed to the total suspended particulates, i.e., particles across the full-size range, cause respiratory and cardiovascular disease^100^ and lung cancer,^101^ with PM_2.5_ carrying the greater risk.^100^ At the lung epithelium, those particles <1 µm could translocate and redistribute via the circulatory system.^94^ For those studies reporting microplastic concentrations in air, 50% used a method with an LOD above 10 µm, with two having an LOD of 50 µm. Four studies (50%) had an LOD of 4 µm or below. Thus, there is a knowledge gap with respect to microplastic particles in the size range considered relevant to human health.

For the tissue studies, one each had a reported instrumental LOD of 1 µm,^42^ 3 µm,^40^ and 5 µm.^44^ Several studies did not report the LOD, and five studies had a limit >20 µm.^33,34,69–71^ Since the likelihood of uptake increases with decreasing size and given the biological plausibility of particle uptake in relation to size as outlined above, it is important that studies are designed with an analytical technique in mind which is compatible with the research question. If this is human exposure, external or internal, efforts toward using techniques with increased spatial resolution should be made. Right now, there is not enough data to understand whether large size ranges can be used as an index to predict amounts in smaller size ranges. Until such studies have been designed and executed, microplastic data in the smaller size ranges is needed.

## Confounding Variables and Challenges

Microplastic analysis presents a plethora of challenges to scientists. As outlined above, a major challenge is improving the methodological and spatial limits of detection, since the smaller the particle, the greater the likelihood of bioavailability in vivo. An obvious limitation is if the method employed involves manual manipulation and transfer of suspected microplastic particles, since these are limited to a size small enough to pick up, for example on a needle. This is usually performed using a stereomicroscope, which allows a user to have their hand in the field of view, e.g., Altunışık^23,52^ and Bordbar et al.^83^ Such microscopes tend to have lower magnification than compound microscopes. Manual transfer most likely occurs if the spectroscopic technique used is ATR FT-IR. Linking back to fit-for-purpose, it is important to ensure the selected analytical technique is optimum for the research question. If this is human exposure, then ATR FT-IR should be avoided as the particle sizes observed are larger than what is considered a concern.

Particle size limits of detection can be improved by using automated and semi-automated techniques, such as imaging and particle-finding software. The latter is more time efficient since the background is not included but some particles may be missed if the optical contrast is poor. However, despite these techniques, the method may also be limited by the pore size of the filter used to retain a sample. For example, Li et al.^55^ used an automated imaging technique, but the methodological LOD was 10 µm due to the pore size of the filter used to retain the sample.

Background fluorescence is another variable which can compromise results.^102^ This is pronounced in Raman spectroscopy, particularly for the 532 nm wavelength laser, which results in higher intensity scattering in the C–H stretching region, but also higher fluorescence. In a study examining the release of microplastic from breast milk storage bags using micro-Raman, the percent of microplastic in the samples could not be estimated due to fluorescence interference with the measurements of small particles.^76^ Using different excitation sources in Raman spectroscopy can lead to variations in the response within specific regions of the Raman spectrum, such as the C–H stretching zone. Guan et al.,^42^ who collected spectral information with a 532 nm excitation wavelength, demonstrated an elevated response in the C–H stretching zone compared to the response obtained with the 785 nm excitation source. This suggests that the choice of excitation source can influence the intensity and sensitivity of certain spectral features, providing researchers with options to optimize their analysis based on the specific regions of interest in the Raman spectrum. Nava et al. recommend the adoption of a 532 nm laser due to enhanced intensity.^102^ However, as previously noted, extra care is needed when classifying spectra acquired with a 532 nm laser, with particular attention being paid to the fingerprint region as the intensity in the C–H stretching region can bias the correlation with reference spectra and thus impact the accuracy of the HQI.

The influence of material degradation on Raman signal to noise has been highlighted as another challenge,^77^ whereby mechanically degraded micro- and nanoplastic particles were not classifiable, and thus micro-Raman measurements were likely an underestimate. In addition, poor spectra may be obtained if the sample or particle is not flat, for example, fibres. Finnegan et al. proposed using the KBr pellet method, but without powder, to press individual particles to a thickness <10 µm.^103^ This sample preparation method, whereby pressed particles remain on the surface of two stainless steel dies, substantially improved the hit quality indices of particles, from 51% to 98% having an HQI > 80%.^103^ Whether this technique could be applicable to small particle sizes by pressing the die onto particulates, rather than manually transferring individual particles, remains to be tested.

Another challenge which researchers face is the spectral correlation between plastic polymers. For example, Levermore et al.^30^ found PE and PA to highly correlate (0.78) when analyzed using Pearson's correlation coefficient. Thus, the threshold for classification had to be set at 0.8 (80%). This also links to potentially confounding non-plastic spectra. For example, in skin, there are characteristic Raman bands at 1240 (amide II), 1298 (phospholipids, collagen), 1448 (CH_2_ bend protein), 1657 (amide I),^104^ and 2930 cm^−1^.^105^ These could interfere with the identification of PA plastics, with characteristic Raman bands in the fingerprint region at 1238 (PA6,6), 1298 (PA6), 1446 (PA6,6), 1448 (PA6), 1643 (PA6, PA6,6), 2882 (PA6,6), and 2942 (PA6).^106^ Thus, extra care is required when interpreting PA spectra, especially for non-fibrous shapes, since skin scales are likely a background contaminant during sample processing and analysis.

Background microplastic contamination is also a key challenge in microplastic analysis, with sources of contamination ranging from laboratory air to apparatus used in protocols. For example, filtration unit lids were identified as the source of background contamination in a study investigating microplastic levels in ground and drinking water,^53^ with 45 microplastic particles contaminating blank samples on average. Levels of background contamination can vary, for example Kirstein et al.^54^ found a minimum of five and maximum of 155 microplastic particles sample^−1^ in procedural blanks (average 46 ± 63). In another example, Thiele et al.^80^ found procedural blanks contained a mean concentration of 9.2 (±3.2) potential microplastic particles filter^−1^. As 84.8% of those were fibres, and 87.0% were transparent/clear, transparent fibres were excluded entirely from the results. On the other hand, Li et al.^63^ found procedural contamination from airborne fibres to be low when analyzing seafood samples, with an average of 0.67 ± 0.75 microplastic particles filter^−1^ detected in the procedural blank samples compared with 8.63 ± 4.35 microplastic particles filter^−1^ for coastal mussel tissues and 5.70 ± 2.27 microplastic particles filter^−1^ for supermarket bought samples. Where possible, field blanks from the sampling environment should also be acquired and analyzed. For example, Ragusa et al.^31^ collected blank samples for a breast milk sampling procedure by rinsing filtered water over the exposed breast dermis. Alternatively, the local atmosphere may be a source of contamination during sampling. Of concern, is the observation of microplastic pollution in the surgical environment. A mean microplastic deposition rate of 5388 ± 2830 microplastic particles m^−2^ day^−1^ during a 12 h working period was reported.^17^ The instrumental LOD was 10 µm, which contradicts the lower LOD reported in a previous publication from the same group using the same instrument.^40^ This may not be an issue for small tissue specimens, which are rapidly collected and covered, or for surgeries excising tissue via keyhole procedures. However, in scenarios where incisions remain open for long periods of time or specimens with large surface areas are sampled, there could be opportunity for background contamination. Thus, when planning studies on human tissue, it is important to include environmental blanks in the operating theatre.

## Recommendations

Based on the presented findings and review, a range of recommendations for future research on microplastic exposure using vibrational spectroscopies can be made:

- New tissue samples are collected with adequate blank samples to monitor potential background contamination from the surgical environment.
- The analytical technique should be fit-for-purpose for the research question
  The spatial resolution should align to the particle sizes of importance for the given matrix.
- Given the risk for background contamination, particularly during sampling, or sample processing, an extra line of evidence would strengthen the case, particularly for human tissue data. Specifically, applying a complimentary spectral imaging technique to qualitatively show the presence of microplastic embedded in tissue sections will support data from digested tissues. Such data would have to conform to a range of inclusion criteria, such as the particle being in the same focal plane as the tissue, away from the edge of the specimen, and away from any red blood cell clusters indicating trauma and hence potential particle introduction via this. It is anticipated that microplastic particles in cells and interstitial tissue will be small, thus enhanced spatial resolution is again recommended.
- Instrumental parameters should be consistently reported across studies
  Laser wavelength, gratings and Raman shift window for Raman spectroscopy, the spectral range analyzed, number of scans and details of background for FT-IR, any spectral pre-processing performed either automatically by software or manually, details of the library and matching threshold or statistical methods used.
- A representative selection of spectra, spanning the range of HQIs accepted, should be presented, clearly showing labelled reference and sample spectra, ideally on the same plot.
- Adequate blanks, and details of how the blanks were treated and used, should be clearly reported.
- The methodological and/or instrumental spatial LOD should be reported if human exposure is the focus. This will help predict absorption, distribution, and elimination, and thus internal dose.
- Particle size and shape, specifically for confirmed microplastic particles, should be reported, and this should be clear. Studies typically mention how many particles have been analyzed, how many are of plastic origin, and a size and shape distribution, however, whether these distributions are particle-wide or microplastic-wide is rarely reported.
- Published evidence should be used to support the conclusions drawn, e.g., to illustrate the biological plausibility and likelihood for particle size and biodistribution in the body when interpreting findings in tissues.

As noted, the field of microplastic pollution and human exposure is growing rapidly. Along with the above recommendations, emerging technologies can help address important knowledge gaps. Advanced hybrid and correlative technologies, coupling or integrating spectroscopy with high resolution microscopy, presents an opportunity for enhanced spatial resolution, lower LOD and therefore the detection of smaller microplastic particles. For example, atomic force microscopy-IR has been used to study the uptake of 500 nm PS spheres into skin fibroblasts.^107^ Correlative SEM and Raman were used to Raman image an individual 300 nm reference microplastic (PS).^108^ However, these techniques will be less widely available, and will still be time intensive, requiring expertise for operation and interpretation of data.

## Conclusion

Microplastic pollutes a range of different environmental matrices which are relevant to human exposure via inhalation or ingestion. To understand the level of risk this exposure presents with respect to the manifestation of hazards in the body, robust data on microplastic concentrations for those particle sizes considered an issue for health are needed. Current data suggests levels of microplastic are low relative to other particles in the exposome. Thus, the potential for uptake and accumulation is of greatest concern as this may build to chronic adverse effects.

Due to its resolved spatial resolution, Raman micro-spectroscopy and imaging are advantageous analytical approaches when investigating human-relevant exposure matrices for microplastic. However, the literature lacks consistency in the reporting of methodological detail, for both IR and Raman techniques, which could otherwise aid comparisons and standardization for future research. Emerging data on internal exposures requires further scrutiny and studies to validate the findings, due to the conflict in the larger particle sizes observed relative to what is expected. Given the risk of background contamination and the importance of such findings, this is both important and responsible. Correlative technologies may provide an opportunity to fill the crucial research questions around plastic particle size distributions.

## Supplemental Material

sj-docx-1-asp-10.1177_00037028231199772 - Supplemental material for Application of Infrared and Near-Infrared Microspectroscopy to Microplastic Human Exposure Measurements Click here for additional data file. Supplemental material, sj-docx-1-asp-10.1177_00037028231199772 for Application of Infrared and Near-Infrared Microspectroscopy to Microplastic Human Exposure Measurements by Stephanie Wright, Joseph Levermore and Yukari Ishikawa in Applied Spectroscopy
