# Supplementary material for: Application of Infrared and Near-Infrared Microspectroscopy to Microplastic Human Exposure Measurements
Source: Appl Spectrosc. 2023 Oct 4;77(10):1105–28. doi: 10.1177/00037028231199772 (PMC10566227; doi:10.1177/00037028231199772)
Supplement: sj-docx-1-asp-10.1177_00037028231199772 - Supplemental material for Application of Infrared and Near-Infrared Microspectroscopy to Microplastic Human Exposure Measurements [file sj-docx-1-asp-10.1177_00037028231199772.docx]

**Supplemental Material**

**Application of Infrared and Near-Infrared Microspectroscopy to Microplastic Human Exposure Measurements**

Stephanie Wright^1,2^*, Joseph Levermore^1^, Yukari Ishikawa^1^

^1^MRC Centre for Environment and Health, School of Public Health, Imperial College London, W12 0BZ, United Kingdom

^2^Health Protection Research Unit in Environmental Exposures and Health, School of Public Health, Imperial College London, W12 0BZ, United Kingdom

*Corresponding email address: s.wright19@imperial.ac.uk

**Table S1.** The references retrieved by the search method and included for the main part of the review.

| **Reference** | **Exposure type** | **Source** | **Source sub-category** |
| --- | --- | --- | --- |
| Hernandez et al.^1^ | Ingestion | Beverage | Tea |
| Altunışık^2^ | Ingestion | Drinking water | Bottled |
| Kankanige and Babel^3^ | Ingestion | Drinking water | Bottled (plastic) |
| Taheri et al.^4^ | Ingestion | Drinking water | Bottled (plastic) |
| Pérez-Guevara et al.^5^ | Ingestion | Drinking water | Kiosk |
| Shruti et al.^6^ | Ingestion | Drinking water | Kiosk |
| Mintenig et al.^7^ | Ingestion | Drinking water | Ground water |
| Kirstein et al.^8^ | Ingestion | Drinking water | Water distribution chain |
| Altunışık^9^ | Ingestion | Beverages | Soft drinks |
| Li et al.^10^ | Ingestion | Drinking water | Bottled |
| Almaiman et al.^11^ | Ingestion | Drinking water | Multiple sources |
| Liu et al.^12^ | Ingestion | Breast milk | Packaging |
| Winkler et al.^13^ | Ingestion | Drinking water | Bottled (plastic) |
| Shi et al.^14^ | Ingestion | Drinking water | Kettle |
| Yin et al.^15^ | Ingestion | Drinking water | Bottled (plastic) |
| Aslam et al.^16^ | Ingestion | Dust | Indoors |
| Liu et al.^17^ | Ingestion | Dust | Indoors |
| Soltani et al.^18^ | Ingestion | Dust | Indoors |
| Quinzi et al.^19^ | Ingestion | Orthodontic aligners |  |
| Sathaish et al.^20^ | Ingestion | Salt |  |
| Ujjaman et al.^21^ | Ingestion | Salt |  |
| Thiele et al.^22^ | Ingestion | Salt |  |
| Bošković et al ^23^ | Ingestion | Seafood | Bivalves |
| Ziino et al.^24^ | Ingestion | Seafood | Processed fish |
| Bordbar et al.^25^ | Ingestion | Seafood | Bivalves |
| Hussien et al.^26^ | Ingestion | Seafood | Processed fish |
| Catarino et al.^27^ | Ingestion | Seafood | Bivalves |
| Li et al.^28^ | Ingestion | Seafood | Bivalves |
| Akoueson et al.^29^ | Ingestion | Seafood |  |
| Imasha and Babel ^30^ | Ingestion | Seafood | Bivalves |
| Afrin et al.^31^ | Ingestion | Sugar |  |
| Vianello et al.^32^ | Inhalation | Indoors | TSP |
| Perera et al.^33^ | Inhalation | Outdoors/Indoors | TSP |
| Amato-Lourenço et al.^34^ | Inhalation | Outdoors | TSP |
| Rahman et al.^35^ | Inhalation | Outdoors | PM2.5 |
| Xie et al.^36^ | Inhalation | Outdoors | TSP |
| Wright et al.^37^ | Inhalation | Outdoors | TSP |
| Levermore et al.^38^ | Inhalation | Outdoors | PM10 |
| Gaston et al.^39^ | Inhalation | Indoors/outdoors | TSP |
| Huang et al.^40^ | Tissue/biofluids | Biofluids | Sputum |
| Qiu et al.^41^ | Tissue/biofluids | Biofluids | BALF |
| Zhao et al.^42^ | Tissue/biofluids | Tissues | Testis |
| Zhu et al.^43^ | Tissue/biofluids | Tissues | Placenta |
| Braun et al.^44^ | Tissue/biofluids | Tissue | Placenta |
| Ibrahim et al.^45^ | Tissue/biofluids | Tissue | Colon |
| Jenner et al.^46^ | Tissue/biofluids | Tissue | Lung |
| Rotchell et al.^47^ | Tissue/biofluids | Tissues | Vein |
| Ragusa et al.^48^ | Tissue/biofluids | Biofluids | Breast milk |
| Guan et al.^49^ | Tissue/biofluids | Biofluids | Enclosed |
| Wu et al.^50^ | Tissue/biofluids | Tissue | Thrombi |
| Amereh et al.^51^ | Tissue/biofluids | Tissues | Placenta |
| Field et al.^52^ |  | Deposition | Indoors |

**References**

1. L.M. Hernandez, E.G. Xu, H.C.E. Larsson, R. Tahara, V.B. Maisuria, N. Tufenkji. “Plastic Teabags Release Billions of Microparticles and Nanoparticles into Tea”. Environ. Sci. Technol. 2019. 53(21): 12300-12310. 10.1021/acs.est.9b02540.

2. A. Altunışık. “Microplastic Pollution and Human Risk Assessment in Turkish Bottled Natural and Mineral Waters”. Environ. Sci. Pollut. Res. Int. 2023. 30(14): 39815-39825. 10.1007/s11356-022-25054-6.

3. D. Kankanige, S. Babel. “Smaller-sized Micro-plastics (MPs) Contamination in Single-use PET-bottled Water in Thailand”. Sci. Total Environ. 2020. 717: 137232. 10.1016/j.scitotenv.2020.137232.

4. S. Taheri, B. Shoshtari-Yeganeh, H. Pourzamani, K. Ebrahimpour. “Investigating the Pollution of Bottled Water by the Microplastics (MPs): the Effects of Mechanical Stress, Sunlight Eposure, and Freezing on MPs Release”. Environ. Monit. Assess. 2022. 195(1): 62. 10.1007/s10661-022-10697-2.

5. F. Pérez-Guevara, P.D. Roy, I. Elizalde-Martínez, G. Kutralam-Muniasamy, V.C. Shruti. “Human Exposure to Mcroplastics from Urban Decentralized Pay-to-fetch Drinking-water Refill Kiosks”. Sci. Total Environ. 2022. 848: 157722. 10.1016/j.scitotenv.2022.157722

6. V.C. Shruti, G. Kutralam-Muniasamy, F. Pérez-Guevara, P.D. Roy, I. Elizalde-Martínez. “Free, but not Microplastic-free, Drinking Water from Outdoor Refill Kiosks: A Challenge and a Wake-up Call for Urban Management”. Environ Poll. 2022. 309: 119800. 10.1016/j.envpol.2022.119800.

7. S.M. Mintenig, M.G.J. Löder, S. Primpke, G. Gerdts. “Low Numbers of Microplastics Detected in Drinking Water from Ground Water Sources”. Sci. Total Environ. 2019. 648: 631-635. 10.1016/j.scitotenv.2018.08.178

8. I.V. Kirstein, F. Hensel, A. Gomiero, L. Iordachescu, A. Vianello, H.B. Wittgren, J. Vollertsen. “Drinking plastics? – Quantification and Qualification of Microplastics in Drinking Water Distribution Systems by µFTIR and Py-GCMS”. Water Res. 2021. 188: 116519. 10.1016/j.watres.2020.116519

9. A. Altunışık. “Prevalence of Microplastics in Commercially Sold Soft Drinks and Human Risk Assessment”. J. Environ. Manage. 2023. 336: 117720. 10.1016/j.jenvman.2023.117720

10. H. Li, L. Zhu, M. Ma, H. Wu, L. An, Z. Yang. “Occurrence of Microplastics in Commercially Sold Bottled Water”. Sci. Total Environ. 2023. 867: 161553. 10.1016/j.scitotenv.2023.161553

11. L. Almaiman, A. Aljomah, M. Bineid, F.M. Aljeldah, F. Aldawsari, B. Liebmann, I. Lomako, K. Sexlinger, R. Alarfaj. “The Occurrence and Dietary Intake Related to the Presence of Microplastics in Drinking Water in Saudi Arabia”. Environ. Monit. Assess. 2021. 193(7): 390. 10.1007/s10661-021-09132-9

12. L. Liu, X. Zhang, P. Jia, S. He, H. Dai, S. Deng, J. Han. “Release of Microplastics From Breastmilk Storage Bags and Assessment of Intake by Infants: A Preliminary Study”. Environ. Poll. 2023. 323: 121197. 10.1016/j.envpol.2023.121197

13. A. Winkler, F. Fumagalli, C. Cella, D. Gilliland, P. Tremolada, A. Valsesia. “Detection and Formation Mechanisms of Secondary Nanoplastic Released from Drinking Water Bottles”. Water Res. 2022. 222: 118848. 10.1016/j.watres.2022.118848

14. Y. Shi, D. Li, L. Xiao, E.D. Sheerin, D. Mullarkey, L. Yang, X. Bai, I.V. Shvets, J.J. Boland, J.J. Wang. “The Influence of Drinking Water Constituents on the Level of Microplastic Release from Plastic Kettles”. J. Hazard. Mater. 2022. 425: 127997. 10.1016/j.jhazmat.2021.127997

15. P.-Y. Lin, I.H. Wu, C.-Y. Tsai, R. Kirankumar, S. Hsieh. “Detecting the Release of Plastic Particles in Packaged Drinking Water Under Simulated Light Irradiation Using Surface-Enhanced Raman spectroscopy”. Anal.Chim. Acta. 2022. 1198: 339516. 10.1016/j.aca.2022.339516

16. I. Aslam, A. Qadir, S.R. Ahmad. “A Preliminary Assessment of Microplastics in Indoor Dust of a Developing Country in South Asia”. Environ. Monit. Assess. 2022. 194(5): 340. 10.1007/s10661-022-09928-3

17. C. Liu, J. Li, Y. Zhang, L. Wang, J. Deng, Y. Gao, L. Yu, J. Zhang, H. Sun. “Widespread Distribution of PET and PC Microplastics in Dust in Urban China and their Estimated Human Exposure”. Environ. Int. 2019. 128: 116-124

18. N.S. Soltani, M.P. Taylor, S.P. Wilson. “Quantification and Exposure Assessment of Microplastics in Australian Indoor House Dust”. Environ. Poll. 2021. 283: 117064. 10.1016/j.envpol.2021.117064

19. V. Quinzi, G. Orilisi, F. Vitiello, V. Notarstefano, G. Marzo, G. Orsini. “A Spectroscopic Study on Orthodontic Aligners: First Evidence of Secondary Microplastic Detachment After Seven Days of Artificial Saliva Exposure”. Sci. Total Environ. 2023. 866: 161356. 10.1016/j.scitotenv.2022.161356

20. M.N. Sathish, I. Jeyasanta, J. Patterson. “Microplastics in Salt of Tuticorin, Southeast Coast of India”. Arch. Environ. Contam. Toxicol. 2020. 79(1): 111-121. 10.1007/s00244-020-00731-0

21. A.-A. Ujjaman Nur, M.B. Hossain, P. Banik, T.R. Choudhury, S.I. Liba, S. Umamaheswari, M.F. Albeshr, V. Senapathi, T. Arai, J. Yu. “Microplastic Contamination in Processed and Unprocessed Sea Salts from a Developing Country and Potential Risk Assessment”. Chemosphere. 2022. 308: 136395

22. C.J. Thiele, L.J. Grange, E. Haggett, M.D. Hudson, P. Hudson, A.E. Russell, L.M. Zapata-Restrepo. “Microplastics in European Sea Salts – An Example of Exposure Through Consumer Choice and of Interstudy Methodological Discrepancies”. Ecotoxicol. Environ. Saf. 2023. 255: 114782. 10.1016/j.ecoenv.2023.114782

23. N. Bošković, D. Joksimović, O. Bajt. “Microplastics in Mussels from the Boka Kotorska Bay (Adriatic Sea) and Impact on Human Health”. Food Chem. Toxicol. 2023. 173: 113641. 10.1016/j.fct.2023.113641

24. G. Ziino, L. Nalbone, F. Giarratana, B. Romano, F. Cincotta, A. Panebianco. “Microplastics in Vacuum Packages of Frozen and Glazed Icefish (Neosalanx spp.): A Freshwater Fish intended for Human Consumption”. Ital.J. Food Saf. 2021. 10(4): 9974. 10.4081/ijfs.2021.9974

25. L. Bordbar, K. Kapiris, S. Kalogirou, A. Anastasopoulou. “First Evidence of Ingested Plastics by a High Commercial Shrimp Species (Plesionika narval) in the Eastern Mediterranean”. Mar. Poll. Bull. 2018. 136: 472-476. 10.1016/j.marpolbul.2018.09.030

26. N.A. Hussien, A. Mohammadein, E.M. Tantawy, Y. Khattab, J.S. Al Malki. “Investigating Microplastics and Potentially Toxic Elements Contamination in Canned Tuna, Salmon, and Sardine fishes from Taif Markets, KSA”. Open Life Sci. 2021. 16(1): 827-837. 10.1515/biol-2021-0086

27. Catarino, A. I., Macchia, V., Sanderson, W. G., Thompson, R. C. & Henry, T. B. Low levels of microplastics (MP) in wild mussels indicate that MP ingestion by humans is minimal compared to exposure via household fibres fallout during a meal.*Environmental Pollution* **237**, 675-684 (2018). https://doi.org:https://doi.org/10.1016/j.envpol.2018.02.069

28. A.I. Catarino, V. Macchia, W.G. Sanderson, R.C. Thompson, T.B. Henry. “Low Levels of Microplastics (MP) in Wild Mussels Indicate that MP Ingestion by Humans is Minimal Compared to Exposure via Household Fibres Fallout During a Meal”. Environ. Poll. 2018. 237: 675-684. 10.1016/j.envpol.2018.02.069

29. F. Akoueson, L.M. Sheldon, E. Danopoulos, S. Morris, J. Hotten, E. Chapman, J. Li, J.M. Rotchell. “A Preliminary Analysis of Microplastics in Edible Versus Non-edible Tissues from Seafood Samples”. Environ. Poll. 2020. 263: 114452. 10.1016/j.envpol.2020.114452

30. H.U.E. Imasha, S. Babel. “Microplastics Contamination in Commercial Green Mussels from Selected Wet Markets in Thailand”. Arch. Environ. Contam. Toxicol. 2021. 81(3): 449-459. 10.1007/s00244-021-00886-4

31. S. Afrin, M.M. Rahman, M.N. Hossain, M.K. Uddin, G. Malafaia. “Are There Plastic Particles in My Sugar? A Pioneering Study on the Characterization of Microplastics in Commercial Sugars and Risk Assessment”. Sci. Total Environ. 2022. 837: 155849. 10.1016/j.scitotenv.2022.155849

32. A. Vianello, R.L. Jensen, L. Liu, J. Vollertsen. “Simulating Human Exposure to Indoor Airborne Microplastics Using a Breathing Thermal Manikin”. Sci. Rep. 2019. 9(1): 8670. 10.1038/s41598-019-45054-w

33. K. Perera, S. Ziajahromi, S. Bengtson Nash, P.M. Manage, F.D.L. Leusch. “Airborne Microplastics in Indoor and Outdoor Environments of a Developing Country in South Asia: Abundance, Distribution, Morphology, and Possible Sources”. Environ. Sci. Technol. 2022. 56(23): 16676-16685. 10.1021/acs.est.2c05885

34. L.F. Amato-Lourenço, N. de Souza Xavier Costa, K.C. Dantas, L. Dos Santos Galvão, F.N. Moralles, S. Lombardi, A.M. Júnior, J.A.L. Lindoso, R.A. Ando, F.G. Lima, R. Carvalho-Oliveira, T. Mauad. “Airborne Microplastics and SARS-CoV-2 in Total Suspended Particles in the Area Surrounding the Largest Medical Centre in Latin America”. Environ. Pollut. 2022. 292(Pt A): 118299. 10.1016/j.envpol.2021.118299

35. L. Rahman, G. Mallach, R. Kulka, S. Halappanavar. “Microplastics and Nanoplastics Science: Collecting and Characterizing Airborne Microplastics in Fine Particulate Matter”. Nanotoxicology. 2021. 15(9): 1253-1278. 10.1080/17435390.2021.2018065

36. Y. Xie, Y. Li, Y. Feng, W. Cheng, Y. Wang. “Inhalable Microplastics Prevails in Air: Exploring the Size Detection Limit”. Environ. Int. 2022. 162: 107151. 10.1016/j.envint.2022.107151

37. S.L. Wright, J.M. Levermore, F.J. Kelly. “Raman Spectral Imaging for the Detection of Inhalable Microplastics in Ambient Particulate Matter Samples”. Environ. Sci. Technol. 2019. 53(15): 8947-8956. 10.1021/acs.est.8b06663

38. J.M. Levermore, T.E.L. Smith, F.J. Kelly, S.L. Wright. “Detection of Microplastics in Ambient Particulate Matter Using Raman Spectral Imaging and Chemometric Analysis”. Anal.Chem. 2020. 92(13): 8732-8740. 10.1021/acs.analchem.9b05445

39. E. Gaston, M. Woo, C. Steele, S. Sukumaran, S. Anderson. “Microplastics Differ Between Indoor and Outdoor Air Masses: Insights from Multiple Microscopy Methodologies”. Appl. Spectrosc. 2020. 74(9): 1079-1098. 10.1177/0003702820920652

40. S. Huang, X. Huang, R. Bi, Q. Guo, X. Yu, Q. Zeng, Z. Huang, T. Liu, H. Wu, Y. Chen, J. Xu, Y. Wu, P. Guo. “Detection and Analysis of Microplastics in Human Sputum”. Environ. Sci. Technol. 2022. 56(4): 2476-2486. 10.1021/acs.est.1c03859

41. L. Qiu, W. Lu, C. Tu, X. Li, H. Zhang, S. Wang, M. Chen, X. Zheng, Z. Wang, M. Lin, Y. Zhang, C. Zhong, S. Li, Y. Liu, J. Liu, Y. Zhou. “Evidence of Microplastics in Bronchoalveolar Lavage Fluid among Never-Smokers: A Prospective Case Series”. Environ. Sci. Technol. 2023. 57(6): 2435-2444. 10.1021/acs.est.2c06880

42. Q. Zhao, L. Zhu, J. Weng, Z. Jin, Y. Cao, H. Jiang, Z. Zhang. “Detection and Characterization of Microplastics in the Human Testis and Semen”. Sci. Total Environ. 2023. 877: 162713. 10.1016/j.scitotenv.2023.162713

43. L. Zhu, J. Zhu, R. Zuo, Q. Xu, Y. Qian, L. An. “Identification of Microplastics in Human Placenta Using Laser Direct Infrared Spectroscopy”. Sci. Total Environ. 2023. 856(Pt 1): 159060. 10.1016/j.scitotenv.2022.159060

44. T. Braun, L. Ehrlich, W. Henrich, S. Koeppel, I. Lomako, P. Schwabl, B. Liebmann. “Detection of Microplastic in Human Placenta and Meconium in a Clinical Setting”. Pharmaceutics. 2021. 13(7): 921.

45. Y.S. Ibrahim, S. Tuan Anuar, A.A. Azmi, W.M.A. Wan Mohd Khalik, S. Lehata, S.R. Hamzah, D. Ismail, Z.F. Ma, A. Dzulkarnaen, Z. Zakaria, N. Mustaffa, S.E. Tuan Sharif, Y.Y. Lee. “Detection of Microplastics in Human Colectomy Specimens”. JGH Open. 2021. 5(1): 116-121. 10.1002/jgh3.12457

46. L.C. Jenner, J.M. Rotchell, R.T. Bennett, M. Cowen, V. Tentzeris, L.R. Sadofsky. “Detection of Microplastics in Human Lung Tissue Using μFTIR Spectroscopy”. Sci. Total Environ. 2022. 831: 154907. 10.1016/j.scitotenv.2022.154907

47. J.M. Rotchell, L.C. Jenner, E. Chapman, R.T. Bennett, I.O. Bolanle, M. Loubani, L. Sadofsky, T.M. Palmer. “Detection of Microplastics in Human Saphenous Vein Tissue Using μFTIR: A Pilot Study”. PLoS One. 2023. 18(2): e0280594. 10.1371/journal.pone.0280594

48. A. Ragusa, V. Notarstefano, A. Svelato, A. Belloni, G. Gioacchini, C. Blondeel, E. Zucchelli, C. De Luca, S. D’Avino, A. Gulotta, O. Carnevali, E. Giorgini. “Raman Microspectroscopy Detection and Characterisation of Microplastics in Human Breastmilk”. Polymers. 2022. 14(13): 2700

49. Q. Guan, J. Jiang, Y. Huang, Q. Wang, Z. Liu, X. Ma, X. Yang, Y. Li, S. Wang, W. Cui, J. Tang, H. Wan, Q. Xu, Y. Tu, D. Wu, Y. Xia. “The Landscape of Micron-Scale Particles Including Microplastics in Human Enclosed Body Fluids”. J. Hazard. Mater. 2023. 442: 130138. 10.1016/j.jhazmat.2022.130138

50. D. Wu, Y. Feng, R. Wang, J. Jiang, Q. Guan, X. Yang, H. Wei, Y. Xia, Y. Luo. “Pigment Microparticles and Microplastics Found in Human Thrombi Based on Raman Spectral Evidence”. J. Adv. Res. 2022. 10.1016/j.jare.2022.09.004

51. F. Amereh, N. Amjadi, A. Mohseni-Bandpei, S. Isazadeh, Y. Mehrabi, A. Eslami, Z. Naeiji, M. Rafiee. “Placental Plastics in Young Women from General Population Correlate with Reduced Foetal Growth in IUGR Pregnancies”. Environ. Poll. 2022. 314: 120174. 10.1016/j.envpol.2022.120174

52. D.T. Field, J.L. Green, R. Bennett, L.C. Jenner, L.R. Sadofsky, E. Chapman, M. Loubani, J.M. Rotchell. “Microplastics in the Surgical Environment”. Environ. Int. 2022. 170: 107630. 10.1016/j.envint.2022.107630
